# Tissue-Specific Metabolite Profiling and Antioxidant Potential of *Eclipta prostrata* Following Different Drying Treatments

**DOI:** 10.3390/ijms27157014

**Published:** 2026-08-04

**Authors:** Amina Bibi, Udomsap Jaitham, Phannika Tongchai, Peerapong Jeeno, Kunrunya Sutan, Sakaewan Ounjaijean, Hataichanok Chuljerm, Anurak Wongta, Sumed Yadoung, Khanchai Danmek, Surat Hongsibsong

**Affiliations:** 1 School of Health Sciences Research, Research Institute for Health Sciences, Chiang Mai University, Chiang Mai 50200, Thailand; amina_bibi@cmu.ac.th (A.B.); udomsap_j@cmu.ac.th (U.J.); phannika.t@cmu.ac.th (P.T.); peerapong_jeen@cmu.ac.th (P.J.); sakaewan.o@cmu.ac.th (S.O.); hataichanok.ch@cmu.ac.th (H.C.); anurak.wongta@cmu.ac.th (A.W.); sumed.yadoung@cmu.ac.th (S.Y.); 2 Environmental, Occupational Health Sciences and Non-Communicable Diseases Center of Excellence, Research Institute for Health Sciences, Chiang Mai University, Chiang Mai 50200, Thailand; kunrunya.s@cmu.ac.th; 3 Biotechnology Program, School of Agriculture and Natural Resources, University of Phayao, Phayao 56000, Thailand; khanchai.da@up.ac.th

**Keywords:** *Eclipta prostrata*, drying methods, freeze drying, LC-QTOF-MS, untargeted metabolomics, antioxidant activity, phenolic compounds, flavonoids, ICP-OES, medicinal plants

## Abstract

*Eclipta prostrata* is a medicinal plant widely used in traditional medicine because of its antioxidant and therapeutic properties; however, comprehensive information on the effects of drying methods on tissue-specific phytochemical composition remains limited. This study evaluated the influence of freeze drying (FD), shade drying (SD), and oven drying (OD) on the antioxidant activity, phenolic and flavonoid contents, elemental composition, and metabolomic profiles of flowers, leaves, roots, and stems of *E. prostrata*. Antioxidant activity was determined using DPPH, ABTS, and FRAP assays; elemental composition was analyzed by ICP-OES; and untargeted metabolomic profiling was performed using LC-QTOF-MS in both positive and negative ionization modes. Freeze-dried samples consistently exhibited the highest antioxidant capacity and retained the greatest total phenolic and flavonoid contents, particularly in flowers, whereas oven drying generally resulted in the greatest reduction in antioxidant activity, especially in roots. ICP-OES analysis demonstrated tissue-dependent differences in mineral composition, with calcium, potassium, magnesium, sodium, iron, zinc, and manganese being the predominant essential elements retained after drying. Untargeted metabolomics revealed tissue-specific metabolic responses to drying, with root tissues exhibiting the greatest metabolomic variation, whereas flowers and stems remained comparatively stable. Multivariate analyses indicated that drying primarily altered the relative abundance of metabolite classes rather than the overall metabolome. The major metabolites detected belonged to flavonoids, coumestans, phenolic acids, lipid-derived metabolites, amino acid derivatives, oxylipins, and triterpenoid/saponin-related compounds, which are associated with the medicinal properties of *E. prostrata*. These findings demonstrate that both plant tissue and drying method significantly influence the retention of bioactive compounds and provide valuable information for optimizing post-harvest processing to preserve the phytochemical quality and therapeutic potential of *E. prostrata*.

## 1. Introduction

Medicinal plants have been an integral part of traditional remedies for centuries, and their use continues to this day due to their therapeutic efficacy and broad pharmacological potential. Synthetic drugs, despite their rapid therapeutic effects, are often associated with adverse side effects that may limit their long-term use and safety in certain populations [1]. As a result, scientific research is increasingly shifting its focus toward medicinal plants. To date, numerous plants have been investigated for their therapeutic potential; those supported by well-established experimental evidence are considered ideal candidates for the future development of pharmaceutical products. Among many prominent names, *Eclipta prostrata* (also known as false daisy), a medicinal plant belonging to the Asteraceae family, has been used traditionally in Ayurvedic, Chinese, and Thai medicine for centuries. It is well known for its anti-inflammatory, antioxidant, antimicrobial, and hepatoprotective properties.

In Thailand, *Eclipta prostrata*, or Kameng, has been traditionally used for treating anemia, tuberculosis, and asthma and also as a blood tonic [2]. Additionally, it was used to self-medicate by AIDS patients and has proven to show inhibitory effects against proteases and integrases of HIV [3]. In Traditional Chinese Medicine, *Eclipta prostrata* (Mo Han Lian) has long been used to treat hemorrhage, tinnitus, premature graying of hair, and other disorders associated with liver and kidney deficiency [4]. *Eclipta prostrata* has also shown various other pharmacological activities, such as antivenom, antimycotic, antitumor, and antihemorrhagic. It is also used to treat hair and skin problems, diarrhea [2] and diphtheria [5].

Building on its diverse pharmacological profile, many of the reported therapeutic effects of *Eclipta prostrata* are closely associated with its ability to modulate oxidative stress [6]. Oxidative stress, which results from an imbalance between reactive oxygen species (ROS) and the antioxidant defense system, plays a significant role in the development of various diseases, including liver disorders, inflammation, and cancer. Given the strong antioxidant potential of *E. prostrata*, further investigations are necessary to optimize its utilization as a safe and effective medicinal resource.

The phytochemical composition and biological activity of medicinal plants can be influenced by both plant part and post-harvest processing conditions. Therefore, this study focuses on evaluating the effects of different drying methods such as freeze, oven, and shade drying on the antioxidant properties and metabolomic profile of *E. prostrata*, while also comparing the distribution and abundance of bioactive compounds among its flowers, leaves, roots, and stems. These three drying methods were selected to represent the most commonly applied post-harvest processing techniques for medicinal plants, encompassing low-temperature preservation (freeze drying), traditional ambient-air drying (shade drying), and heat-assisted drying (oven drying). These methods differ markedly in drying temperature, duration, and moisture-removal mechanisms, allowing a systematic comparison of their effects on the retention of antioxidant activity, mineral composition, and tissue-specific metabolite profiles in *E. prostrata*. To the best of our knowledge, no study has concurrently investigated all four plant parts under these drying conditions in combination with untargeted metabolomic profiling. Identifying the most suitable drying treatment and the plant part with the greatest bioactive potential may contribute to the development of standardized processing strategies and improve the quality of *E. prostrata*-based pharmaceutical and nutraceutical products.

## 2. Results

### 2.1. Antioxidant Activity

The antioxidant capacity of the flower, leaf, root and stem of *Eclipta prostrata* using different drying methods was assessed through DPPH, ABTS and FRAP assays. The differences among drying methods and plant parts were statistically significant (*p* < 0.05).

#### 2.1.1. DPPH Radical Scavenging Activity

The DPPH activity of *Eclipta prostrata* was significantly influenced by both plant part and drying methods (Table 1). Two-way ANOVA revealed significant effects of drying method (F (2,24) = 77.81, *p* < 0.001), plant part (F (3,24) = 8.54, *p* < 0.001), and their interaction (F (6,24) = 23.28, *p* < 0.001) on DPPH IC_50_, indicating that the influence of drying method depended on the plant tissue.

Freeze-drying showed the lowest IC_50_ values (mg/mL), indicating stronger scavenging activity compared to other drying treatments. Oven-dried samples showed the lowest scavenging activity, whereas shade-dried samples produced intermediate effects. Across all treatments, freeze-dried flowers showed the greatest scavenging power; however, oven-dried roots produced the weakest effect.

#### 2.1.2. ABTS Radical Scavenging Activity

A similar trend was also observed in the ABTS assay (Table 1). Two-way ANOVA revealed significant effects of drying method (F (2,24) = 130.63, *p* < 0.001), plant part (F (3,24) = 75.51, *p* < 0.001), and their interaction (F (6,24) = 75.38, *p* < 0.001) on ABTS IC_50_, indicating that the effect of drying treatment varied among plant tissues.

The freeze-dried samples preserved ABTS radical scavenging activity significantly across all plant parts, as demonstrated by the consistently lower IC_50_ values (mg/mL), especially freeze-dried flowers, which demonstrated the highest antioxidant activity. In contrast, oven-dried roots demonstrated the weakest activity across all samples. Shade-dried samples, on the other hand, showed intermediate activity compared to other treatments.

#### 2.1.3. Ferric Reducing Antioxidant Power (FRAP)

The ferric reducing antioxidant power of *E. prostrata* extracts was also significantly affected by the drying method and plant part (Table 1). Two-way ANOVA revealed significant effects of drying method (F (2,24) = 233.17, *p* < 0.001), plant part (F (3,24) = 23.44, *p* < 0.001), and their interaction (F (6,24) = 14.28, *p* < 0.001) on FRAP values, indicating that the influence of drying treatment on ferric reducing antioxidant power differed among plant tissues.

Freeze drying resulted in the highest reducing capacity across all plant parts, whereas oven and shade drying markedly reduced FRAP values. A consistently better ferric reducing power was observed in all flower samples regardless of drying method. These findings indicate that freeze drying was the most effective method for preserving the reducing capacity of *E. prostrata* extracts.

### 2.2. Total Phenolic and Flavonoid Content

**Figure 1:**
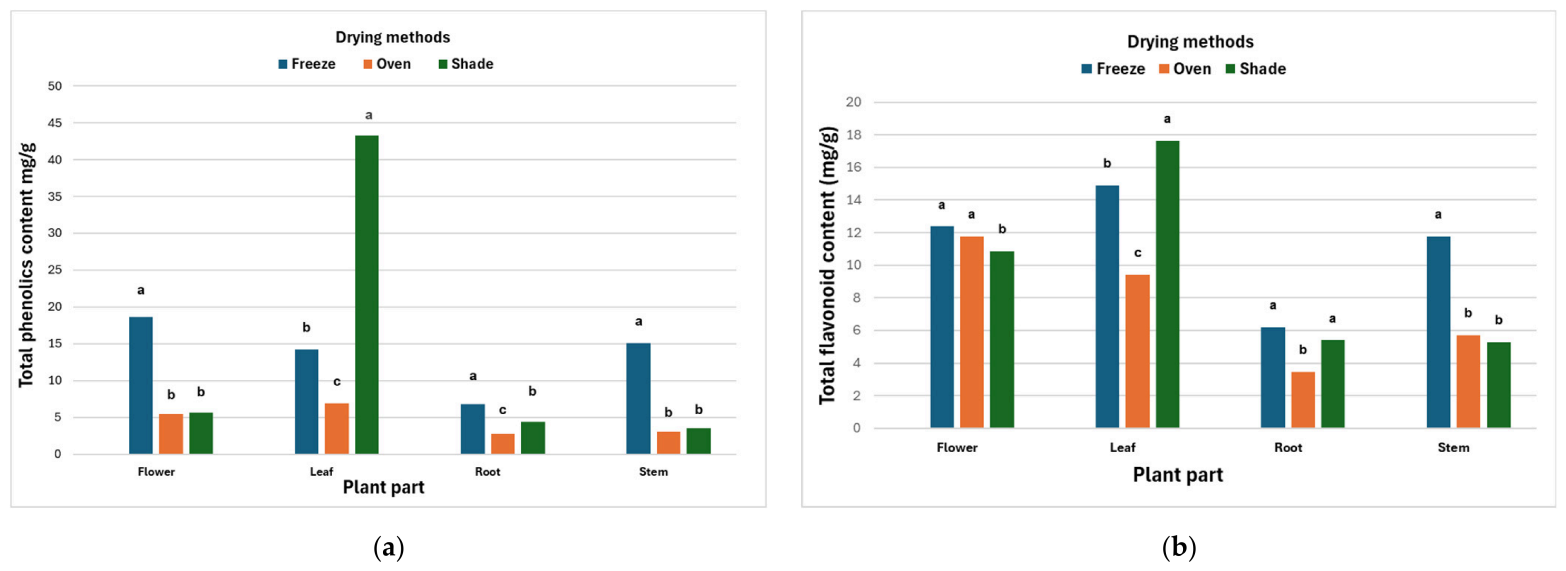
(**a**) Total phenolic content (TPC) and (**b**) total flavonoid content (TFC). Different lowercase letters above the bars within the same plant part indicate significant differences among drying methods according to Tukey’s HSD test (*p* < 0.05).

TPC and TFC values are presented in Figure 1. The total phenolic content (TPC) (Figure 1a) of *Eclipta prostrata* extracts varied significantly among plant parts and drying methods. Two-way ANOVA revealed significant effects of drying method (F (2,24) = 670.56, *p* < 0.001), plant part (F (3,24) = 942.18, *p* < 0.001), and their interaction (F (6,24) = 684.11, *p* < 0.001) on total phenolic content (TPC), indicating that the influence of drying treatment on phenolic accumulation differed among plant tissues.

Among all treatments, shade-dried leaves (43.30 ± 0.53 ^a^) exhibited the highest TPC, followed by freeze-dried flowers (18.63 ± 0.52 ^a^) and stems (15.10 ± 1.06 ^a^), whereas no significant differences were observed between shade- and oven-dried flowers or stems (*p* > 0.05). Oven-dried roots showed the lowest phenolic content (2.81 ± 0.06 ^c^). Overall, freeze drying was more effective at preserving phenolic compounds in flowers, roots, and stems, while shade drying produced the greatest phenolic retention in leaves.

The total flavonoid content (TFC) was also significantly influenced by both plant part and drying method, with a significant interaction detected between these factors (Figure 1b). Two-way ANOVA demonstrated significant effects of drying method (F (2,24) = 107.51, *p* < 0.001), plant part (F (3,24) = 372.74, *p* < 0.001), and their interaction (F (6,24) = 49.64, *p* < 0.001) on total flavonoid content (TFC), indicating that flavonoid retention following drying was tissue-dependent. Shade-dried leaves contained the highest TFC (17.63 ± 0.55 ^a^), which was significantly higher than freeze- and oven-dried leaves. In flowers, freeze- and oven-dried samples exhibited comparable TFC values, both of which were significantly higher than shade-dried flowers. Similarly, freeze- and shade-dried roots showed no significant difference, but both had significantly higher TFC than oven-dried roots. For stems, freeze drying produced the highest TFC, whereas shade- and oven-dried stems did not differ significantly. These results indicate that the optimal drying method for flavonoid preservation varied according to plant part, with shade drying being most effective for leaves and freeze drying generally providing higher flavonoid retention in the remaining tissues.

### 2.3. Inductively Coupled Plasma Optical Emission Spectrometry (ICP-OES) Analysis

ICP-OES analysis revealed the presence of macronutrients, such as calcium (Ca), K, Mg, Na, and P (Table 2); micro-minerals B, Cu, Fe, Mn, and Zn (Table 3); and some heavy metals As, Cd, Co, Cr, Ni, Pb, Al, Ba, V and Si (Table 4) in *Eclipta prostrata*. Within macronutrients, calcium was the most abundant, with shade-dried stems showing its highest concentration, which was followed by freeze-dried stems. Potassium and magnesium were found in all samples, where stems and leaves exhibited overall higher abundance as compared to flowers and roots. Sodium was most elevated in leaves, especially in oven-dried samples, whereas phosphorus was highest in freeze-dried flowers. Significant differences in macronutrient concentrations were observed among drying methods for all plant parts (*p* < 0.05).

Among the micro-minerals, iron showed the highest abundance in shade-dried roots, followed by freeze-dried roots; however, oven-dried roots exhibited significantly lower concentrations (*p* < 0.05). Boron, copper, manganese, and zinc were detected in all plant parts, although their concentrations differed significantly among drying methods. Shade drying resulted in significantly higher boron concentrations in leaves, roots, and stems, whereas oven drying produced the highest boron concentration in flowers. Copper did not show significant differences among flower samples but was significantly more abundant in shade-dried roots and stems than roots and stems that underwent other drying treatments (*p* < 0.05). Manganese concentrations were highest in shade-dried leaves, roots, and stems, while freeze- and shade-dried flowers were significantly higher in manganese concentrations than oven-dried flowers. Zinc concentrations showed tissue-dependent distribution, with oven drying producing the highest concentrations in flowers and leaves, whereas shade drying resulted in significantly higher zinc concentrations in roots and stems, and freeze-dried roots and shade-dried flowers were enriched in silicon.

Heavy metal analysis demonstrated that arsenic, cadmium, cobalt, chromium, nickel, lead, aluminum, barium and vanadium were either absent or present in relatively low concentrations in most plant parts. Significant differences in arsenic concentration were observed only in roots, where shade-dried samples had significantly higher concentrations than freeze-dried and oven-dried roots (*p* < 0.05), whereas no significant differences were detected among drying treatments in flowers, leaves, or stems. Cadmium concentrations were generally low and frequently below the detection limit; however, shade-dried stems and root samples contained the highest detectable concentrations. Chromium was detected in oven-dried flowers, all leaf samples, shade-dried roots, and shade-dried stems, with significant differences observed among drying methods in leaves and roots. Nickel and lead concentrations were notable in roots, with shade-dried roots exhibiting significantly higher concentrations of nickel and lead than freeze-dried roots, while both elements were not detected in oven-dried roots. Aluminum concentrations were highest in shade-dried roots, while oven-dried flowers and leaves contained significantly higher aluminum concentrations than the corresponding freeze- and shade-dried samples. Barium concentrations were found to be the highest in shade-dried roots. Vanadium was detected in oven-dried flowers, all leaf samples, and roots, with shade-dried roots exhibiting the highest concentration, significantly exceeding freeze-dried roots and oven-dried roots, where vanadium was not detected (*p* < 0.05). Overall, the highest concentrations of 6.45 ppm As, 10.01 ppm Cr, 1.56 ppm Pb, 1.36 ppm Ni, and 3.90 ppm V were observed in shade-dried roots. The arsenic and chromium concentrations exceeded the maximum permissible limits of 5 ppm and 2 ppm established by the World Health Organization (WHO) for medicinal plants [7], whereas the elevated chromium concentration indicates a potential toxicological concern because of its known adverse health effects. Although the detected concentrations of other heavy metals remained below the permissible limits, the accumulation of toxic metals in shade-dried roots suggests that this processing method may increase elemental contamination and should therefore be carefully considered when selecting raw materials for medicinal applications.

### 2.4. LC-QTOF-MS Metabolomic Profiling of Eclipta Prostrata

Untargeted LC-QTOF-MS analysis revealed a variety of metabolites in both positive and negative ionization modes in all samples of *Eclipta prostrata.* The multivariate analysis was done using MetaboAnalyst 6.0. Compounds with a score ≥ 90 were selected from positive ionization mode results, and for negative ionization mode, a threshold of >80 was selected due to a smaller number of detected compounds. This criterion was adopted because negative electrospray ionization generally detects fewer metabolite features than positive ionization and to retain biologically relevant metabolites for comparative multivariate analysis while maintaining acceptable annotation confidence.

Most of the prominent chemical classes belonged to lipids, fatty acids, phenolic and flavonoid, and other secondary metabolites. All the results are presented in sequence, where positive ionization mode interpretations are presented first, followed by those from negative mode.

#### 2.4.1. Multivariate Analysis

Principal component analysis (PCA) evaluated different metabolic profiles among the drying groups: FD, OD and SD.

**Figure 2:**
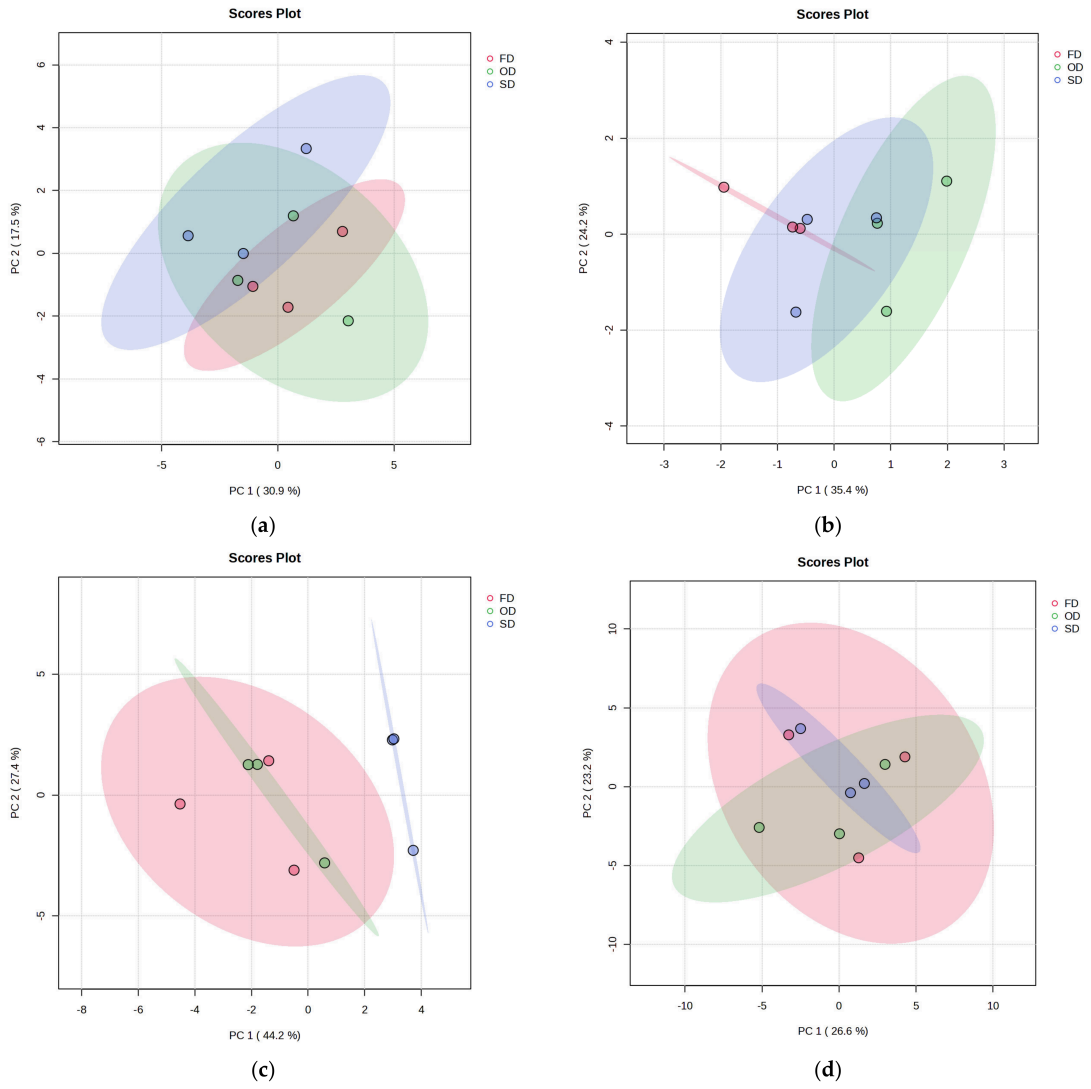
Principal component analysis (PCA) score plots of the untargeted LC–QTOF–MS metabolomic profiles of *Eclipta prostrata* extracts acquired in positive ionization mode following different drying treatments. Score plots are shown for (**a**) flower, (**b**) leaf, (**c**) root, and (**d**) stem tissues. Each point represents an individual biological replicate, and the ellipses indicate the 95% confidence interval for each treatment group. FD, freeze-dried; OD, oven-dried; SD, shade-dried. Background colors denote the three sample groups: FD, OD, and SD and their distribution in PCA space.

The flower samples showed considerable overlap among the three drying treatments. The first two principal components explained 48.4% of the total variance, with PC1 accounting for 30.9% and PC2 accounting for 17.5% (Figure 2a). The PCA score plot revealed substantial overlap among the confidence ellipses of the FD, OD, and SD groups, indicating generally similar metabolite profiles regardless of drying treatment. Although the SD samples exhibited greater dispersion than the FD and OD groups, no distinct treatment-specific clustering was observed. Pairwise PERMANOVA supported these observations, revealing negligible differences between FD and OD (R^2^ = 0.001, *p* = 1.00) and relatively small effect sizes between FD and SD (R^2^ = 0.294, *p* = 0.40) and OD and SD (R^2^ = 0.243, *p* = 0.20). After adjustment for multiple comparisons, all pairwise comparisons remained non-significant (padj = 0.60–1.00), indicating that drying treatment had minimal influence on the overall metabolomic profile of flower tissues.

The leaf samples exhibited moderate separation among the drying treatments, with the first two principal components explaining 59.6% of the total variance (PC1 = 35.4%, PC2 = 24.2%) (Figure 2b). FD samples clustered predominantly on the negative side of PC1, whereas OD samples were distributed mainly on the positive side. SD samples occupied an intermediate position and partially overlapped with both FD and OD, suggesting moderate treatment-related differences in metabolite composition. Pairwise PERMANOVA indicated the greatest variation between FD and OD (R^2^ = 0.573, *p* = 0.10), whereas smaller effect sizes were observed for FD and SD (R^2^ = 0.294, *p* = 0.20) and OD and SD (R^2^ = 0.252, *p* = 0.50). However, none of the comparisons remained statistically significant after adjustment for multiple testing (padj = 0.30–0.50), indicating that the observed clustering trends should be interpreted with caution.

The root samples displayed the clearest separation among the drying treatments. The first two principal components explained 71.6% of the total variance, with PC1 accounting for 44.2% and PC2 accounting for 27.4% (Figure 2c). SD samples formed a distinct cluster on the positive side of PC1 with minimal overlap with the other treatments, whereas FD samples were clearly separated on the negative side of PC1. OD samples occupied an intermediate position between FD and SD. These clustering patterns indicate that root metabolite composition was strongly influenced by the drying method. Pairwise PERMANOVA further supported these observations, showing a very low effect size between FD and OD (R^2^ = 0.057, *p* = 0.70) but substantially larger effect sizes for FD and SD (R^2^ = 0.580, *p* = 0.10) and OD and SD (R^2^ = 0.498, *p* = 0.10). Although all comparisons remained non-significant following adjustment for multiple comparisons (padj = 0.15), the consistently large effect sizes between SD and the other treatments suggest that the root metabolome was the most responsive tissue to drying conditions in positive ionization mode.

In contrast, the stem samples showed extensive overlap among the three drying treatments despite the first two principal components explaining 49.8% of the total variance (PC1 = 26.6%, PC2 = 23.2%) (Figure 2d). FD samples exhibited the greatest within-group dispersion, whereas SD samples formed a relatively compact cluster near the center of the score plot. OD samples were distributed between the FD and SD groups with substantial overlap of the confidence ellipses, indicating generally similar metabolomic profiles across the three drying treatments. Pairwise PERMANOVA further supported this observation, producing low effect sizes between FD and OD (R^2^ = 0.062, *p* = 0.80), FD and SD (R^2^ = 0.027, *p* = 1.00), and OD and SD (R^2^ = 0.138, *p* = 0.70). After adjustment for multiple comparisons, all pairwise comparisons remained non-significant (padj = 1.00), indicating that drying treatment exerted minimal influence on the metabolomic profile of stem tissues.

**Figure 3:**
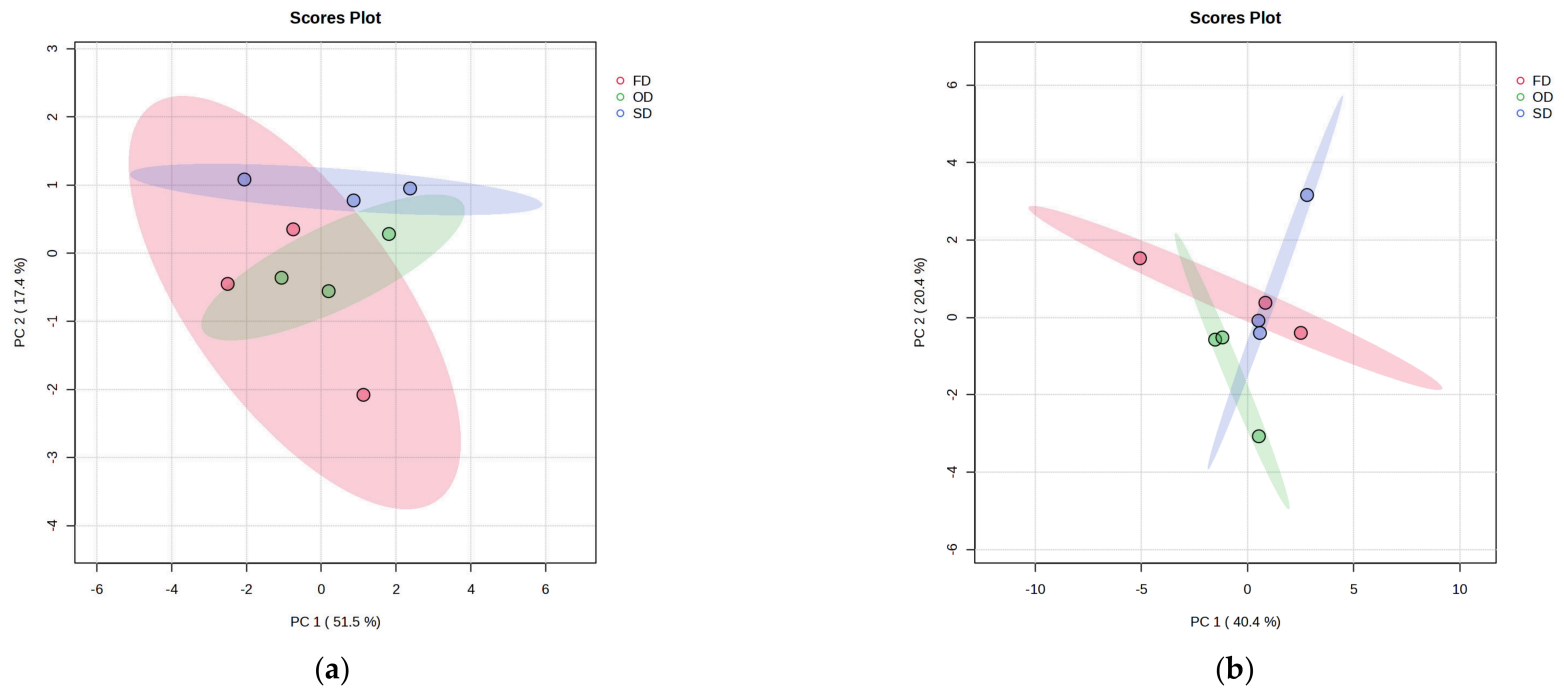

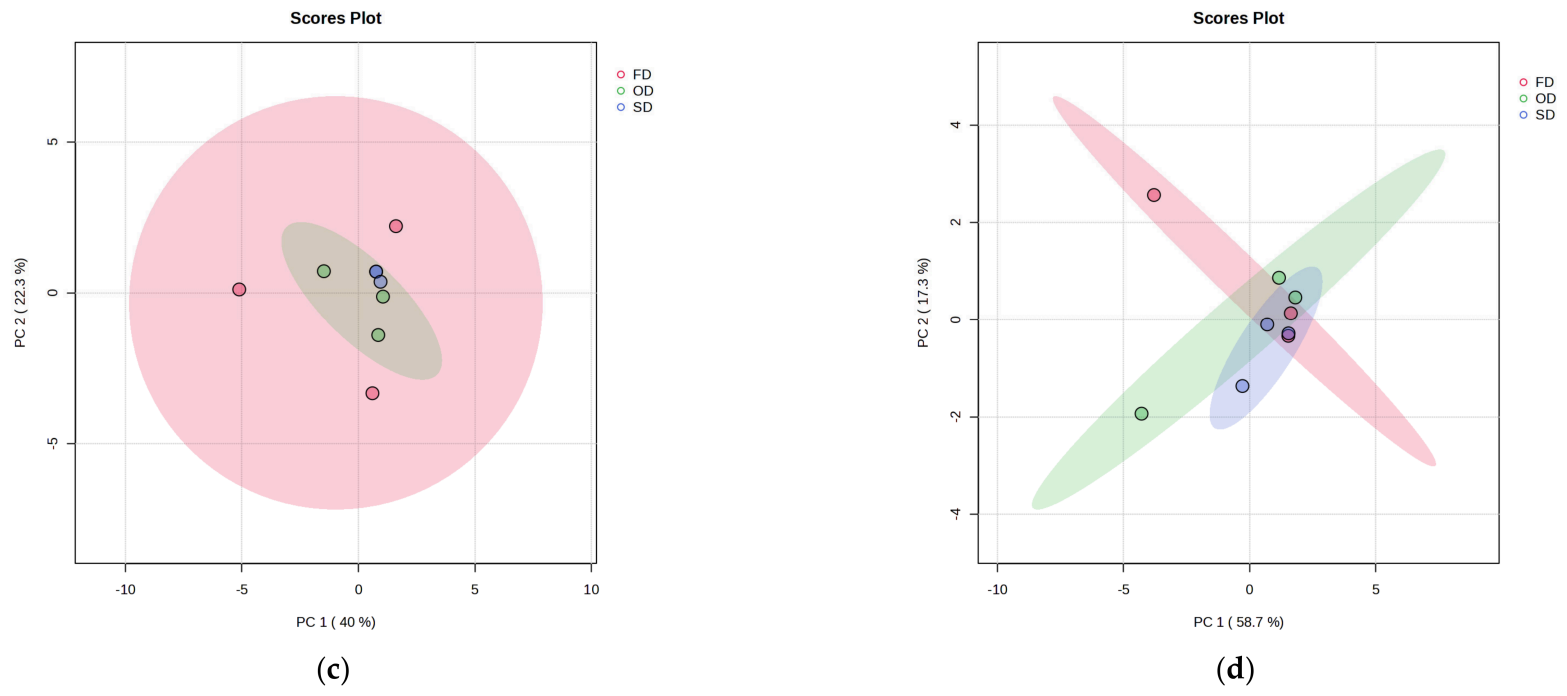
Principal component analysis (PCA) score plots of the untargeted LC–QTOF–MS metabolomic profiles of *Eclipta prostrata* extracts acquired in negative ionization mode following different drying treatments. Score plots are shown for (**a**) flower, (**b**) leaf, (**c**) root, and (**d**) stem tissues. Each point represents an individual biological replicate, and the ellipses indicate the 95% confidence interval for each treatment group. FD, freeze-dried; OD, oven-dried; SD, shade-dried. Background colors denote the three sample groups: FD, OD, and SD and their distribution in PCA space.

In negative ionization mode, the flower samples exhibited substantial overlap among the three drying treatments. The first two principal components explained 68.9% of the total variance, with PC1 accounting for 51.5% and PC2 accounting for 17.4% (Figure 3a). The PCA score plot showed considerable overlap among the confidence ellipses of the FD, OD, and SD groups, indicating generally similar metabolite profiles irrespective of the drying treatment. Although the FD samples displayed greater within-group dispersion than the OD and SD groups, no distinct treatment-specific clustering was observed. Pairwise PERMANOVA supported these observations, revealing low effect sizes between FD and OD (R^2^ = 0.122, *p* = 0.70), FD and SD (R^2^ = 0.231, *p* = 0.40), and OD and SD (R^2^ = 0.118, *p* = 0.60). After adjustment for multiple comparisons, all pairwise comparisons remained non-significant (padj = 0.70), indicating that drying treatment had little influence on the overall metabolomic profile of flower tissues.

The leaf samples exhibited moderate separation among the drying treatments, with the first two principal components explaining 60.8% of the total variance (PC1 = 40.4%, PC2 = 20.4%) (Figure 3b). FD samples were distributed primarily along the negative side of PC1, whereas OD samples clustered mainly on the positive side of PC1. SD samples occupied an intermediate position and partially overlapped with both FD and OD, although one SD sample was clearly separated along PC2. These clustering patterns suggest moderate treatment-related differences in metabolite composition. Pairwise PERMANOVA indicated the greatest variation between OD and SD (R^2^ = 0.439, *p* = 0.10), whereas comparatively smaller effect sizes were observed for FD and OD (R^2^ = 0.118, *p* = 0.60) and FD and SD (R^2^ = 0.109, *p* = 0.90). However, none of the comparisons remained statistically significant after adjustment for multiple testing (padj = 0.30–0.90), suggesting that the observed clustering trends should be interpreted with caution.

The root samples explained 62.3% of the total variance, with PC1 accounting for 40.0% and PC2 accounting for 22.3% (Figure 3c). FD samples exhibited greater within-group dispersion, whereas OD and SD samples clustered closely together, with substantial overlap of their confidence ellipses. Although one FD sample was separated toward the negative side of PC1, the remaining samples were located near the OD and SD clusters, indicating generally similar metabolomic profiles across the drying treatments. Consistent with the PCA, pairwise PERMANOVA produced low effect sizes between FD and OD (R^2^ = 0.037, *p* = 1.00), FD and SD (R^2^ = 0.127, *p* = 1.00), and OD and SD (R^2^ = 0.222, *p* = 0.40). After adjustment for multiple comparisons, all pairwise comparisons remained non-significant (padj = 1.00), indicating that drying treatment exerted minimal influence on the root metabolome in negative ionization mode.

The stem samples exhibited the highest proportion of explained variance among the tissues, with PC1 accounting for 58.7% and PC2 accounting for 17.3%, resulting in a cumulative variance of 76.0% (Figure 3d). OD and SD samples clustered closely near the center of the score plot with substantial overlap, whereas FD samples showed greater dispersion due to one sample positioned on the negative side of PC1. Despite this variation, the confidence ellipses of the three drying treatments overlapped extensively, indicating generally similar metabolomic profiles among the stem tissues. Pairwise PERMANOVA further supported these observations, producing very low effect sizes between FD and OD (R^2^ = 0.029, *p* = 1.00), FD and SD (R^2^ = 0.128, *p* = 0.90), and OD and SD (R^2^ = 0.063, *p* = 0.90). After adjustment for multiple comparisons, all pairwise comparisons remained non-significant (padj = 1.00), indicating that drying treatment had minimal impact on the stem metabolome.

#### 2.4.2. Heatmap Analysis

Hierarchical clustering analysis (HCA) in positive ionization mode further illustrated tissue-specific differences in metabolite abundance among the three drying treatments. Overall, the clustering patterns were generally consistent with the PCA results, indicating that the response of the metabolome to drying varied among different plant tissues.

**Figure 4:**
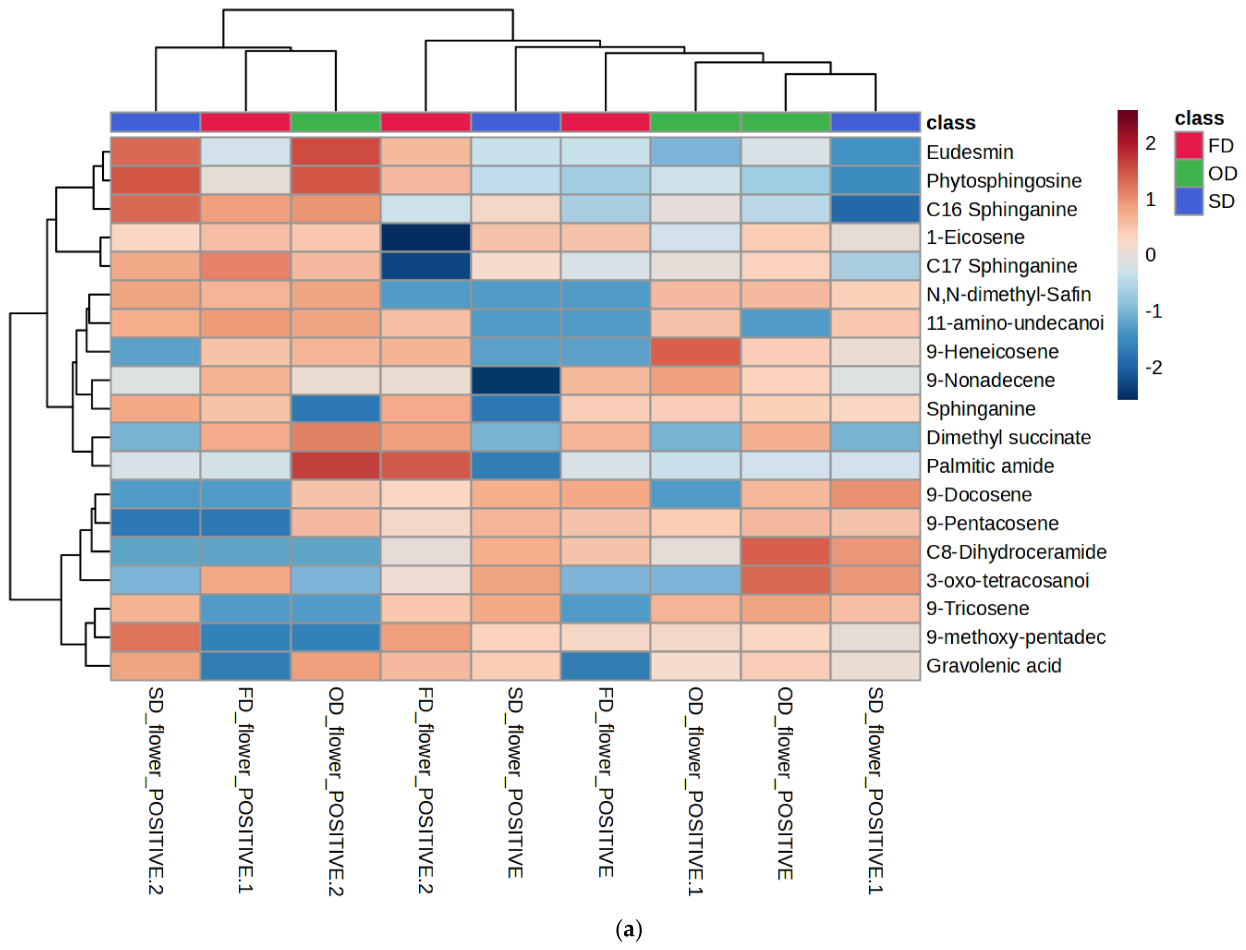

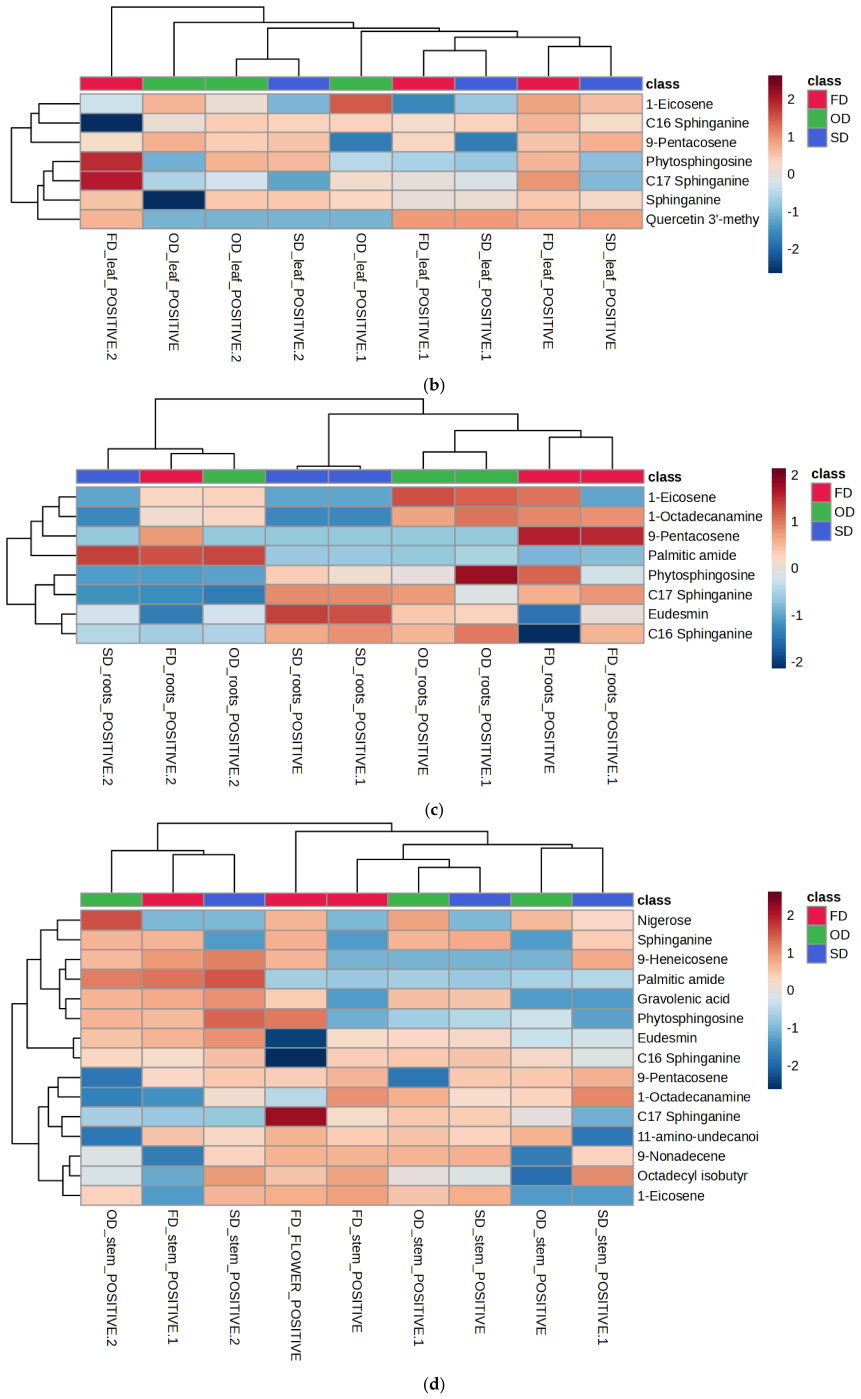
Hierarchical clustering heatmaps of the untargeted LC–QTOF–MS metabolomic profiles of *Eclipta prostrata* extracts acquired in positive ionization mode following different drying treatments. Heatmaps are shown for (**a**) flower, (**b**) leaf, (**c**) root, and (**d**) stem tissues. Samples were clustered based on Pearson’s correlation (*r*) similarity. FD, freeze-dried; OD, oven-dried; SD, shade-dried.

The flower samples exhibited substantial overlap among FD, OD, and SD treatments, with no clear treatment-specific clustering pattern (Figure 4a). Although variation in the relative abundance of several metabolites was observed among individual samples, the overall metabolite profiles remained broadly similar across the three drying methods. The metabolites contributing to the observed variation included the *Eclipta*-associated lignan eudesmin, together with tentatively annotated sphingolipids, fatty acid derivatives, amino acid derivatives, and terpenoid-related metabolites. These results suggest that the flower metabolome was relatively stable regardless of the drying method.

The leaf samples displayed moderate clustering according to drying treatment, although partial overlap among the groups remained evident (Figure 4b). FD replicates showed relatively greater similarity to one another, whereas OD and SD samples exhibited moderate variation in metabolite abundance. The observed differences were primarily associated with the relative abundance of tentatively annotated sphingolipids, fatty acid derivatives, and the flavonoid quercetin 3′-methyl ether, together with the *Eclipta*-associated lignan eudesmin. Overall, these findings indicate that drying influenced the abundance of selected metabolite classes rather than causing substantial alterations to the overall metabolomic profile.

The root samples exhibited the clearest treatment-dependent clustering among all tissues (Figure 4c). FD, OD, and SD formed relatively distinct groups, indicating that root metabolites were the most responsive to drying conditions. The metabolites contributing to this separation included eudesmin, together with tentatively annotated sphingolipids, fatty acid derivatives, and amino acid derivatives. These clustering patterns suggest that drying treatments differentially affected the relative abundance of multiple metabolite classes in root tissues, consistent with the greater separation observed in the PCA.

In contrast, the stem samples showed considerable overlap among the three drying treatments despite moderate variation in the abundance of individual metabolites (Figure 4d). No consistent treatment-specific clustering pattern was evident, indicating relatively minor changes in metabolite composition. The detected metabolites were dominated by eudesmin, together with tentatively annotated sphingolipids, fatty acid derivatives, amino acid derivatives, and terpenoid-related metabolites. These findings suggest that stem tissues were comparatively less affected by the different drying methods than root tissues.

Overall, hierarchical clustering analysis in positive ionization mode demonstrated a tissue-dependent response to drying treatments. Root tissues exhibited the greatest metabolomic variation, whereas flower and stem tissues maintained comparatively stable metabolite profiles across the different drying methods. The observed differences were mainly associated with changes in the relative abundance of the *Eclipta*-associated metabolite eudesmin together with tentatively annotated lipid-related and secondary metabolite classes, rather than the appearance or disappearance of unique metabolites. Because most metabolite identities were assigned based on accurate-mass matching, these annotations should be regarded as putative and require confirmation by MS/MS fragmentation or authentic reference standards.

**Figure 5:**
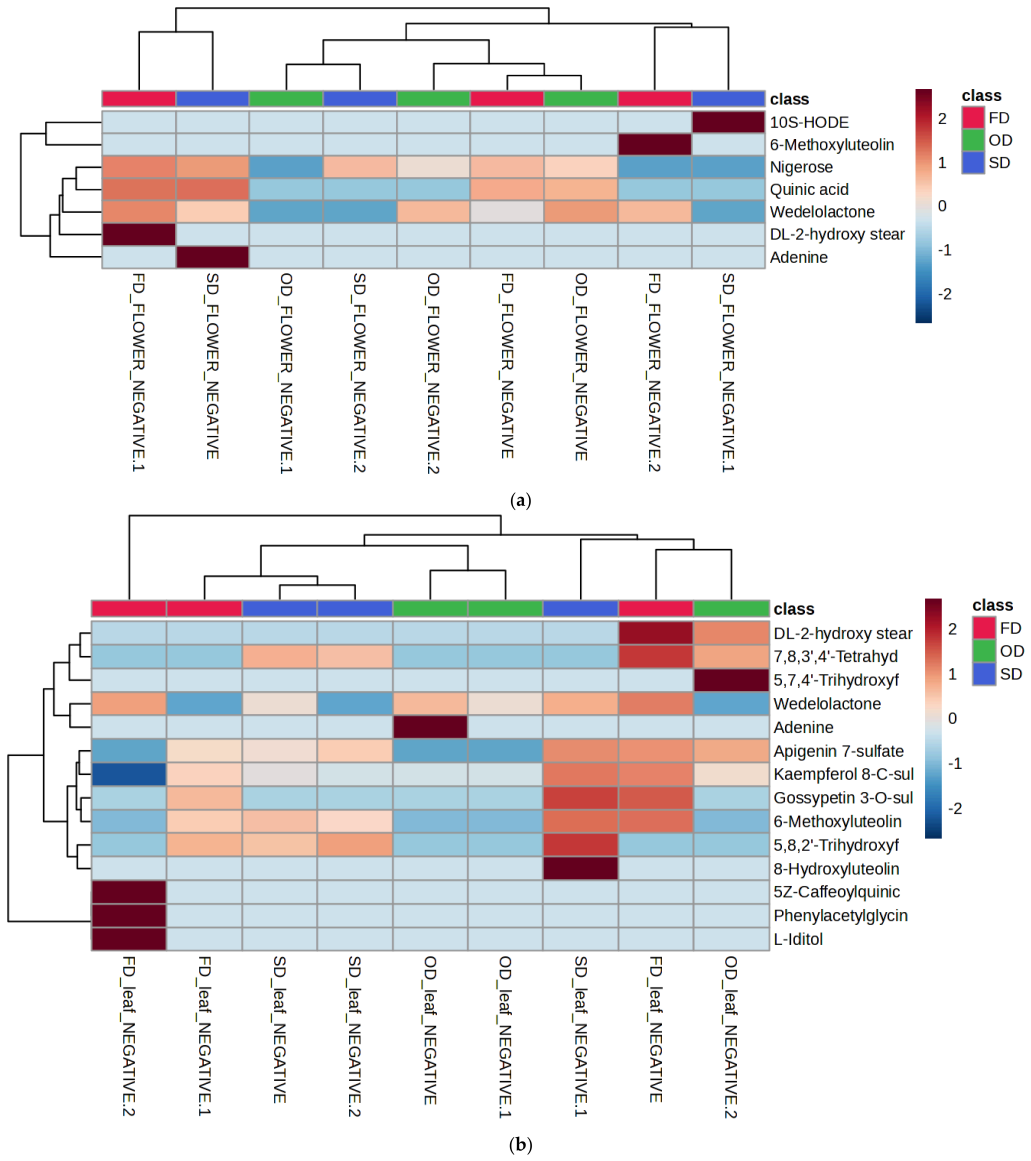

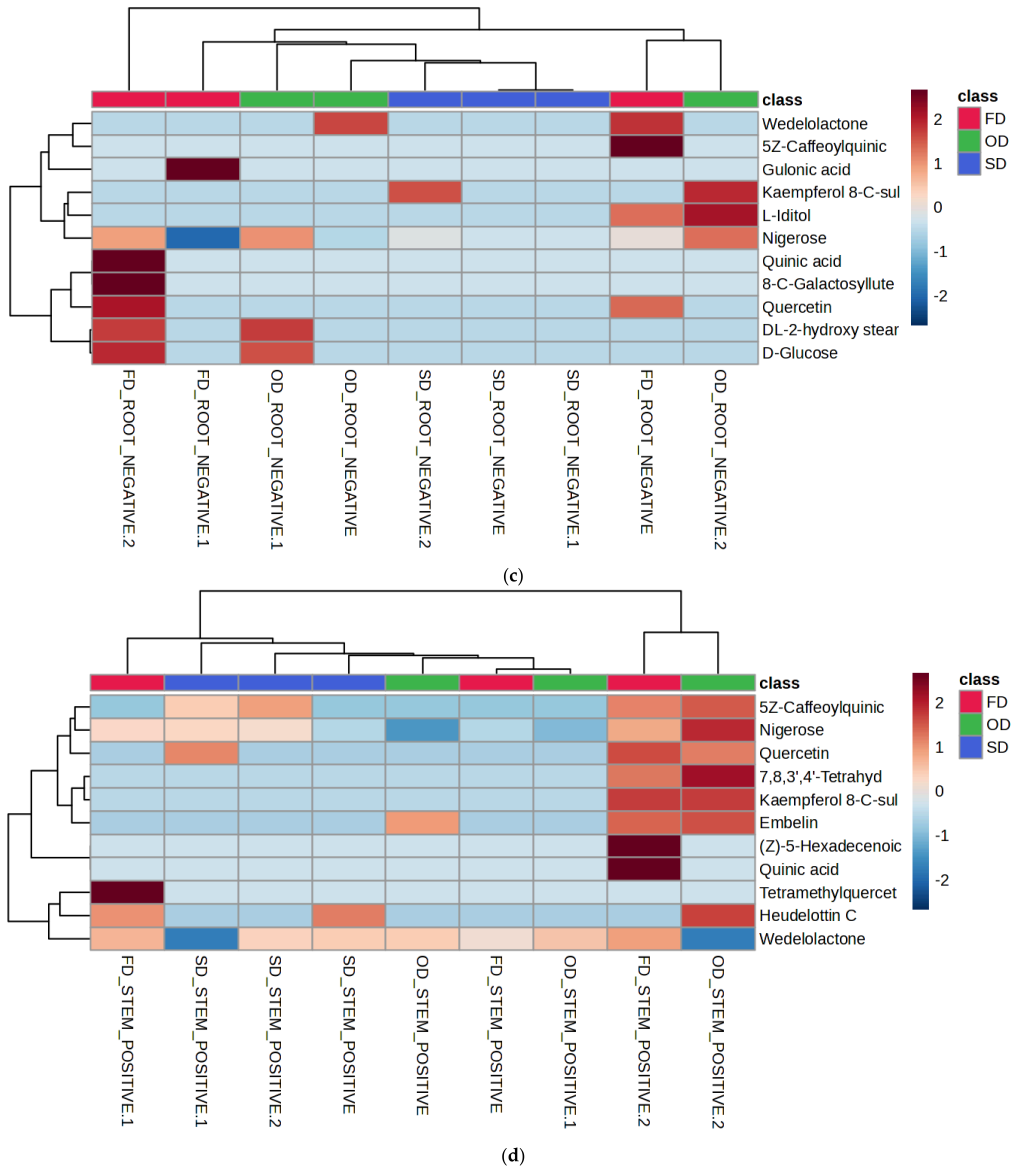
Hierarchical clustering heatmaps of the untargeted LC–QTOF–MS metabolomic profiles of *Eclipta prostrata* extracts acquired in negative ionization mode following different drying treatments. Heatmaps are shown for (**a**) flower, (**b**) leaf, (**c**) root, and (**d**) stem tissues. Samples were clustered based on Pearson’s correlation (*r*) similarity. FD, freeze-dried; OD, oven-dried; SD, shade-dried.

Hierarchical clustering analysis (HCA) in negative ionization mode revealed tissue-specific differences in metabolite abundance among the three drying treatments (Figure 5). Overall, the clustering patterns were generally consistent with the PCA results, indicating that the response of the metabolome to drying varied among different plant tissues.

The flower samples exhibited substantial overlap among FD, OD, and SD treatments, with no distinct treatment-specific clustering pattern (Figure 5a). Although several metabolites varied in relative abundance among individual samples, the overall metabolite profiles remained broadly similar across the three drying methods. The observed variation was primarily associated with the *Eclipta*-associated metabolites wedelolactone and 6-methoxyluteolin, together with tentatively annotated carbohydrates, fatty acid derivatives, purine derivatives, and hydroxylated fatty acid metabolites. These findings suggest that the flower metabolome remained relatively stable regardless of the drying treatment.

The leaf samples exhibited moderate clustering according to drying treatment, although partial overlap among the confidence groups remained evident (Figure 5b). FD replicates showed relatively greater similarity, whereas OD and SD samples displayed moderate variation in metabolite abundance. The differential abundance was mainly associated with the *Eclipta*-associated metabolites wedelolactone and 6-methoxyluteolin, together with tentatively annotated flavonoid sulfates, phenolic acid derivatives, fatty acid derivatives, and carbohydrate metabolites. Overall, these results indicate that drying influenced the relative abundance of selected metabolite classes without causing pronounced alterations to the global metabolomic profile.

The root samples displayed the clearest treatment-dependent clustering among all tissues (Figure 5c). FD, OD, and SD formed relatively distinct groups, indicating that root metabolites were the most responsive to drying conditions. The metabolites contributing to this separation included the *Eclipta*-associated metabolites wedelolactone and quercetin, together with tentatively annotated flavonoid sulfates, phenolic acid derivatives, sugar alcohols, carbohydrates, and fatty acid derivatives. These findings suggest that drying treatments differentially affected the preservation or relative abundance of multiple metabolite classes in root tissues, consistent with the greater separation observed in the PCA.

In contrast, the stem samples exhibited considerable overlap among the three drying treatments despite moderate variation in the abundance of individual metabolites (Figure 5d). No consistent treatment-specific clustering pattern was evident, indicating relatively minor changes in metabolite composition. The detected metabolites included the *Eclipta*-associated metabolites wedelolactone, quercetin, and embelin, together with tentatively annotated flavonoid derivatives, phenolic acid derivatives, fatty acid derivatives, and carbohydrate metabolites. These observations suggest that stem tissues were comparatively less affected by drying than root tissues.

Overall, hierarchical clustering analysis in negative ionization mode demonstrated a tissue-dependent response to drying treatments. Root tissues exhibited the greatest metabolomic variation, whereas flower and stem tissues maintained comparatively stable metabolite profiles across the different drying methods. The observed differences were mainly associated with changes in the relative abundance of *Eclipta*-associated metabolites, including wedelolactone, quercetin, embelin, and 6-methoxyluteolin, together with tentatively annotated flavonoids, phenolic acid derivatives, fatty acid derivatives, and carbohydrate-related metabolites. Because most metabolite identities were assigned based on accurate-mass matching, these annotations should be regarded as putative and require confirmation by MS/MS fragmentation or authentic reference standards.

#### 2.4.3. Correlation Analysis

Correlation heatmaps of all samples showed many negative and positive correlated clusters of several compounds.

Correlation analysis was performed to evaluate the relationships among the discriminative metabolites identified in different tissues under positive ionization mode. Overall, the correlation matrices revealed tissue-specific co-abundance patterns, with several metabolites forming distinct correlation clusters that likely reflect shared biochemical pathways or coordinated responses to drying treatments.

**Figure 6:**
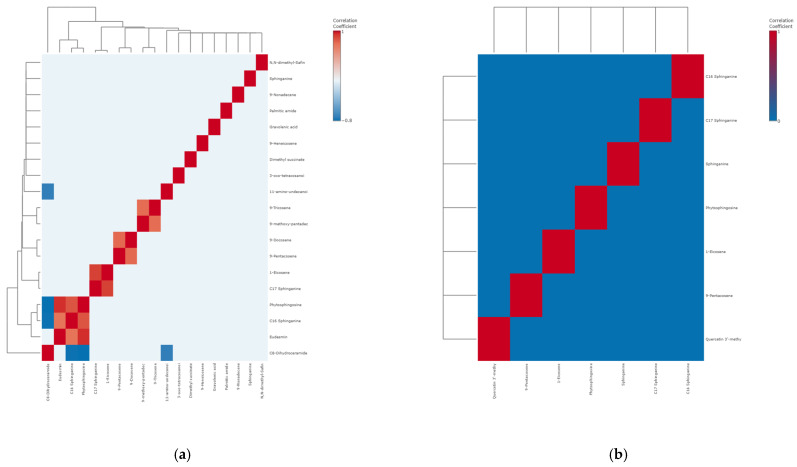

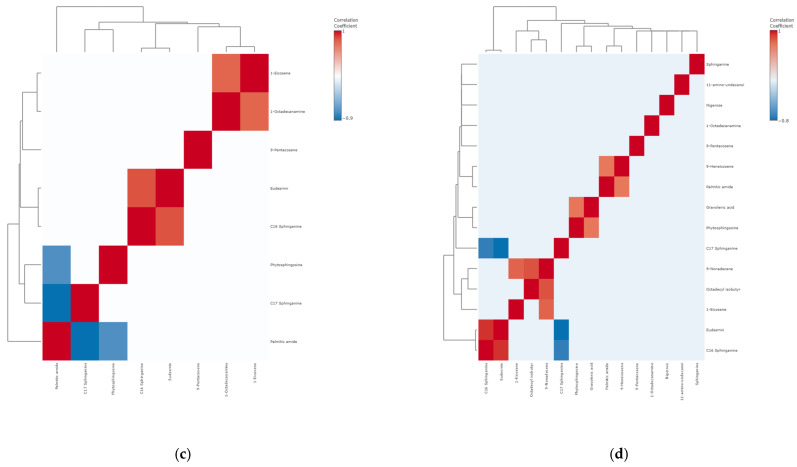
Correlation maps for positive-mode samples: (**a**) flower, (**b**) leaf, (**c**) root, and (**d**) stem.

The flower samples exhibited several positively correlated metabolite groups (Figure 6a). The strongest positive correlations were observed among tentatively annotated sphingolipids, including sphinganine-related metabolites and phytosphingosine, whereas eudesmin showed positive associations with these lipid-related metabolites. Additional positive correlations were observed among tentatively annotated fatty acid derivatives and long-chain hydrocarbons, while relatively few negative correlations were detected. These findings suggest coordinated regulation of lipid-associated metabolites within flower tissues.

The leaf samples displayed comparatively simple correlation patterns, with most metabolites showing weak or negligible associations (Figure 6b). Positive correlations were primarily observed among tentatively annotated sphingolipid metabolites, whereas quercetin 3′-methyl ether exhibited limited correlation with the remaining metabolites. Overall, the relatively weak correlation structure indicates that the discriminative metabolites in leaf tissues responded largely independently to the drying treatments.

The root samples exhibited more pronounced metabolite associations than the other tissues (Figure 6c). Strong positive correlations were observed among tentatively annotated sphingolipids, whereas the *Eclipta*-associated lignan eudesmin was positively correlated with several lipid-related metabolites. In addition, a cluster of tentatively annotated fatty acid derivatives showed coordinated abundance patterns. Conversely, several negative correlations were observed between sphingolipid-related metabolites and fatty-acid-associated metabolites, suggesting differential regulation of these metabolite groups in response to drying.

The stem samples also demonstrated several positively correlated metabolite clusters (Figure 6d). The strongest associations occurred among tentatively annotated sphingolipids, fatty acid derivatives, and long-chain hydrocarbon metabolites. The *Eclipta*-associated lignan eudesmin showed positive correlations with several sphingolipid-related metabolites but weaker associations with other metabolite classes. Overall, the correlation network of stem tissues indicated coordinated variation among lipid-related metabolites despite the limited treatment-specific differences observed in PCA and hierarchical clustering analyses.

Overall, correlation analysis in positive ionization mode demonstrated that tentatively annotated sphingolipids and fatty acid-related metabolites constituted the major co-varying metabolite groups across tissues, whereas *Eclipta*-associated metabolites, including eudesmin, exhibited tissue-dependent relationships with these metabolite clusters. Because metabolite identification was based primarily on accurate-mass matching, these biochemical relationships should be regarded as putative and require further confirmation by MS/MS fragmentation and authentic reference standards.

Correlation analysis was performed to investigate the relationships among the discriminative metabolites detected in negative ionization mode. Overall, the correlation matrices revealed tissue-specific co-abundance patterns, with several metabolite clusters exhibiting coordinated variation that may reflect shared biosynthetic pathways or common responses to the drying treatments.

**Figure 7:**
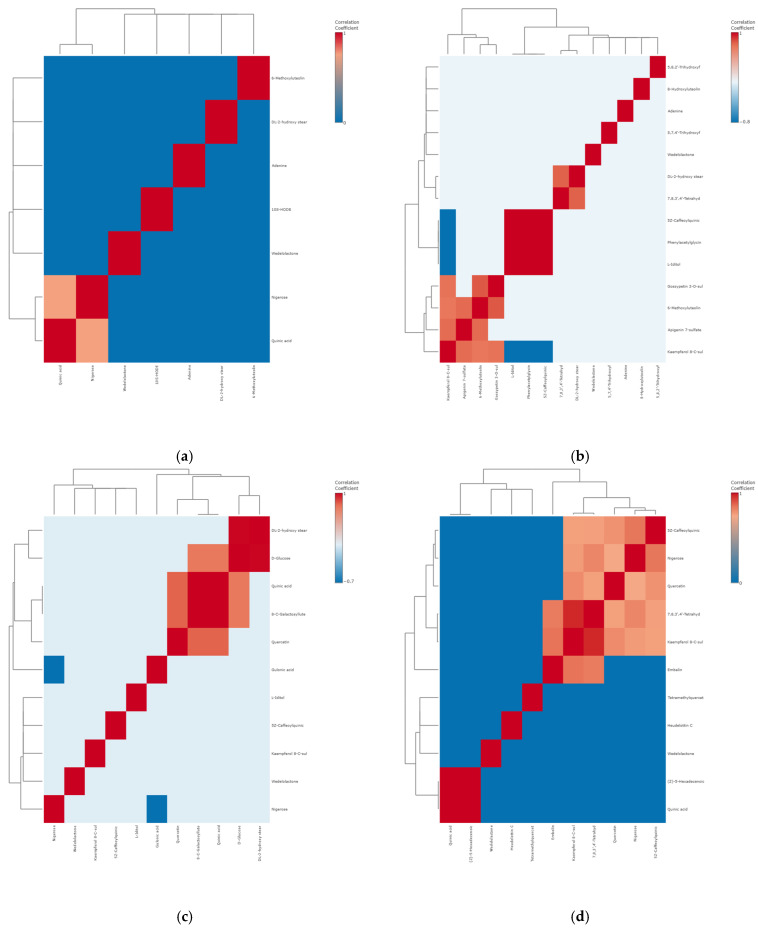
Correlation maps for negative ionization-mode samples: (**a**) flower, (**b**) leaf, (**c**) root, and (**d**) stem.

The flower samples displayed relatively simple correlation patterns, with most metabolites exhibiting weak or negligible associations (Figure 7a). A positive correlation was observed between the carbohydrate-related metabolites quinic acid and tentatively annotated disaccharides, whereas the remaining metabolites showed limited relationships with one another. The *Eclipta*-associated metabolites wedelolactone and 6-methoxyluteolin exhibited minimal correlation with other detected metabolites, suggesting that these compounds varied independently within flower tissues.

The leaf samples exhibited more complex correlation patterns than the flower samples (Figure 7b). Strong positive correlations were observed among the *Eclipta*-associated metabolites 6-methoxyluteolin and wedelolactone, together with tentatively annotated flavonoid derivatives, phenolic acid derivatives, and carbohydrate-related metabolites. In contrast, weak negative correlations were observed between several flavonoid-related metabolites and carbohydrate-associated compounds. These findings indicate coordinated variation among phenolic metabolites in leaf tissues following different drying treatments.

The root samples showed distinct clusters of positively correlated metabolites (Figure 7c). The *Eclipta*-associated metabolite quercetin exhibited positive correlations with tentatively annotated flavonoid glycosides, carbohydrate-related metabolites, and phenolic acid derivatives, whereas wedelolactone displayed comparatively weaker associations with the remaining metabolites. Several carbohydrate-associated metabolites also formed a tightly correlated cluster, suggesting coordinated regulation of primary and secondary metabolism within root tissues.

The stem samples exhibited the strongest correlation network among the tissues, with several metabolites forming highly interconnected clusters (Figure 7d). Strong positive correlations were observed among the *Eclipta*-associated metabolites quercetin and embelin, together with tentatively annotated flavonoid derivatives and phenolic-acid-related metabolites. Wedelolactone showed weaker associations with these metabolite groups, whereas several carbohydrate-related metabolites were positively correlated with flavonoid-derived compounds. These results suggest coordinated accumulation of phenolic metabolites in stem tissues despite the relatively limited separation observed in the multivariate analyses.

Overall, correlation analysis in negative ionization mode demonstrated that *Eclipta*-associated phenolic metabolites, including wedelolactone, quercetin, and 6-methoxyluteolin, exhibited tissue-dependent relationships with tentatively annotated flavonoid derivatives, phenolic acid derivatives, and carbohydrate-related metabolites. The strongest metabolite associations were observed in root and stem tissues, whereas flower tissues displayed comparatively weaker metabolite correlations. Because metabolite identities were assigned primarily by accurate-mass matching, these metabolite annotations and their inferred biochemical relationships should be regarded as putative, pending confirmation by MS/MS fragmentation or authentic reference standards.

#### 2.4.4. PLS-DA

Partial Least Squares Discriminant Analysis (PLS-DA) identified several metabolites contributing significantly to discrimination among drying treatments and plant tissues. Due to the tentative nature of metabolite annotation in untargeted LC-MS analysis, compounds with unusual occurrence in plant systems were treated as discriminatory features and were not used for biological interpretation without further MS/MS confirmation.

Because the number of biological replicates for the flower samples was insufficient to generate a robust PLS-DA model, VIP analysis was performed only for the leaf, root, and stem tissues.

**Figure 8:**
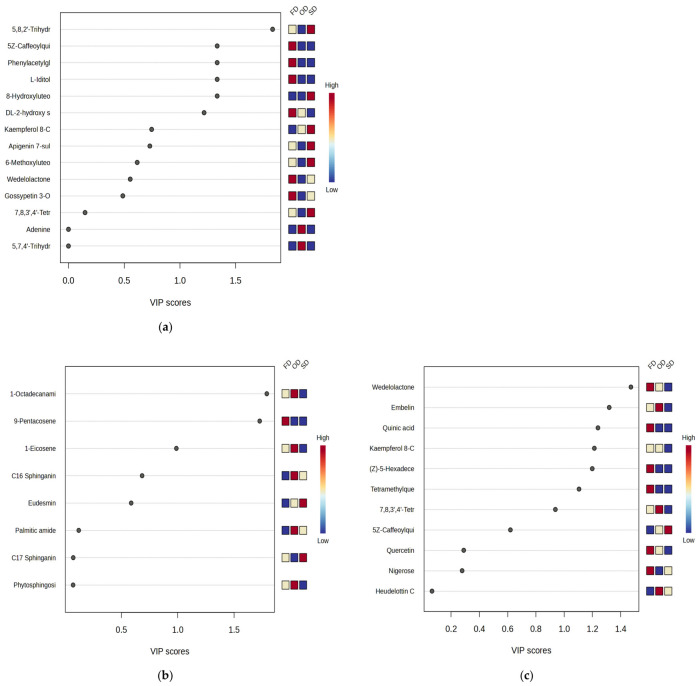
Variable importance in projection (VIP) score plots identifying the metabolites that contributed most to the discrimination among drying treatments in the untargeted LC–QTOF–MS metabolomic analysis of *Eclipta prostrata* extracts acquired in positive ionization mode. VIP score plots are shown for (**a**) leaf, (**b**) root, and (**c**) stem tissues.

The leaf samples were primarily discriminated by tentatively annotated long-chain fatty amines, long-chain hydrocarbons, sphingolipids, and the *Eclipta*-associated lignan eudesmin (Figure 8a). The highest VIP scores were observed for tentatively annotated octadecanamine and long-chain hydrocarbon metabolites, followed by C16 sphinganine and eudesmin, indicating that these metabolites contributed most strongly to the differentiation among drying treatments. The heatmap accompanying the VIP plot further indicated that several lipid-related metabolites accumulated preferentially in the OD treatment, whereas eudesmin exhibited relatively higher abundance in SD samples.

The root samples exhibited the largest number of discriminative metabolites (Figure 8b). The metabolites with the highest VIP scores included tentatively annotated amino acid derivatives, sphingolipids, and the *Eclipta*-associated lignan eudesmin. Additional contributors included tentatively annotated long-chain hydrocarbons, fatty acid derivatives, and carbohydrate-related metabolites. The abundance patterns suggested that several lipid-associated metabolites were enriched in the FD treatment, whereas eudesmin showed comparatively higher abundance under SD, indicating that drying methods differentially influenced the accumulation of these metabolite classes in root tissues.

The stem samples also displayed several metabolites with high discriminatory power (Figure 8c). The most influential metabolites included tentatively annotated amino acid derivatives, C16 sphinganine, and the *Eclipta*-associated lignan eudesmin, together with tentatively annotated long-chain hydrocarbons, fatty acid derivatives, and carbohydrate-related metabolites. Although numerous metabolites contributed to treatment discrimination, most exhibited moderate VIP scores, suggesting that the observed differences among drying treatments resulted from the combined effects of multiple metabolite classes rather than a few dominant compounds.

Overall, VIP analysis in positive ionization mode demonstrated that tentatively annotated sphingolipids, fatty acid-derived metabolites, and amino acid derivatives were the principal contributors to metabolomic variation among drying treatments, whereas the *Eclipta*-associated metabolite eudesmin consistently ranked among the important discriminative metabolites across tissues. These findings further support the tissue-dependent metabolic responses to drying observed in the PCA and hierarchical clustering analyses. Because metabolite identities were assigned primarily by accurate-mass matching, these metabolites should be regarded as putatively annotated pending confirmation by MS/MS fragmentation or authentic reference standards.

Variable importance in projection (VIP) analysis was performed to identify the metabolites contributing most to the separation among drying treatments in negative ionization mode. Because the number of biological replicates for the flower samples was insufficient to generate a reliable PLS-DA model, VIP analysis was performed only for the leaf, root, and stem tissues.

**Figure 9:**
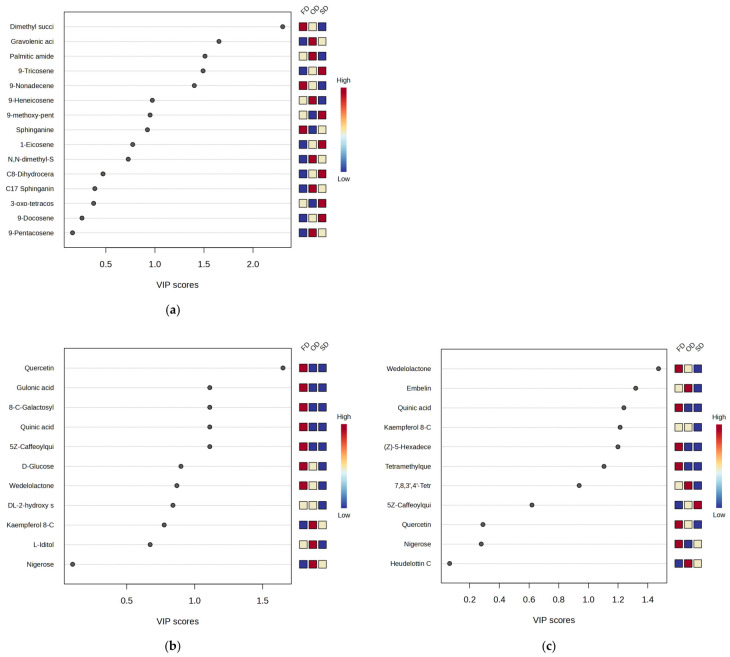
Variable importance in projection (VIP) score plots identifying the metabolites that contributed most to the discrimination among drying treatments in the untargeted LC–QTOF–MS metabolomic analysis of *Eclipta prostrata* extracts acquired in negative ionization mode. VIP score plots are shown for (**a**) flower, (**b**) root, and (**c**) stem tissues.

The leaf samples were primarily discriminated by tentatively annotated fatty acid derivatives, phenolic acid derivatives, flavonoid derivatives, and carbohydrate-related metabolites (Figure 9a). The highest VIP scores were observed for tentatively annotated dimethyl succinate and gravolenic acid, followed by fatty acid amides and long-chain hydrocarbon metabolites. Overall, the results indicate that lipid-associated metabolites contributed most strongly to the separation among drying treatments in leaf tissues.

The root samples were characterized by several discriminative metabolites belonging to both primary and secondary metabolic pathways (Figure 9b). The *Eclipta*-associated metabolite quercetin exhibited the highest VIP score, followed by tentatively annotated carbohydrate-related metabolites, including gulonic acid, quinic acid, and 5Z-caffeoylquinic acid, together with wedelolactone and tentatively annotated flavonoid glycosides. These findings suggest that both phenolic metabolites and carbohydrate-associated metabolites contributed substantially to treatment differentiation in root tissues.

The stem samples were characterized by several discriminative metabolites belonging to both primary and secondary metabolic pathways (Figure 9c). The *Eclipta*-associated metabolites wedelolactone and embelin exhibited the highest VIP scores, followed by quinic acid and tentatively annotated flavonoid derivatives, including kaempferol 8-C-sulfate, 7,8,3′,4′-tetrahydroxyflavone, and tetramethylquercetin derivatives, together with 5Z-caffeoylquinic acid and tentatively annotated fatty acid derivatives. These findings suggest that both phenolic metabolites and flavonoid-related metabolites, together with carbohydrate- and fatty-acid-associated metabolites, contributed substantially to treatment differentiation in stem tissues.

Overall, VIP analysis in negative ionization mode demonstrated that *Eclipta*-associated phenolic metabolites, particularly quercetin, wedelolactone, and embelin, together with tentatively annotated flavonoid derivatives, phenolic acid derivatives, carbohydrate-related metabolites, and fatty acid derivatives, were the principal contributors to metabolomic variation among drying treatments. These results further support the tissue-dependent metabolic responses observed in the PCA, hierarchical clustering, and correlation analyses. As the metabolite identities were assigned primarily by accurate-mass matching, these annotations should be regarded as putative, pending confirmation by MS/MS fragmentation or authentic reference standards.

#### 2.4.5. Metabolite Enrichment Analysis

**Figure 10:**
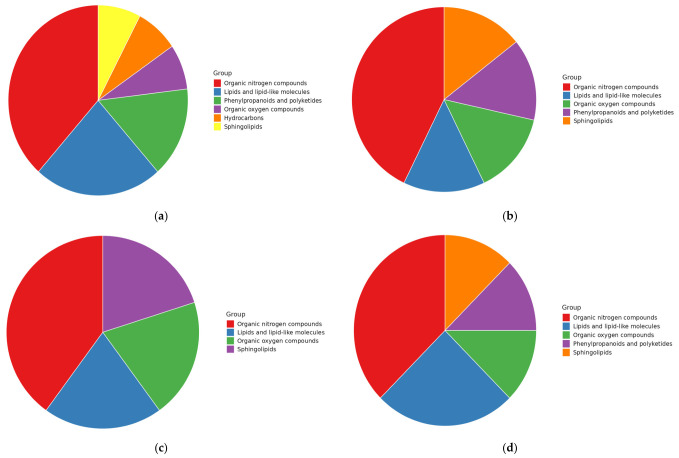
Metabolite distribution in *Eclipta prostrata* under positive ionization mode: (**a**) flower, (**b**) leaf, (**c**) root, and (**d**) stem. The inner ring represents superclasses, and the outer ring represents subclasses of the identified metabolites.

The positive ionization mode revealed distinct metabolite class distributions among the different tissues of *Eclipta prostrata*. Flower extracts were predominantly composed of lipids and lipid-like molecules, followed by organic acids and their derivatives, benzenoids, sphingolipids, organic oxygen compounds, and organic nitrogen compounds (Figure 10a). In contrast, leaf extracts were characterized by a higher proportion of phenylpropanoids and polyketides, together with lipids and lipid-like molecules, organic oxygen compounds, benzenoids, and sphingolipids (Figure 10b). Root extracts exhibited a relatively simple metabolite class composition, consisting mainly of phenylpropanoids and polyketides, lipids and lipid-like molecules, organic oxygen compounds, and benzenoids (Figure 10c). Similarly, stem extracts were dominated by phenylpropanoids and polyketides and lipids and lipid-like molecules, with smaller contributions from benzenoids, organic oxygen compounds, and sphingolipids (Figure 10d). Overall, lipids and lipid-like molecules, together with phenylpropanoids and polyketides, represented the predominant metabolite classes detected across the different tissues of *E. prostrata*, although their relative abundances varied among plant parts.

**Figure 11:**
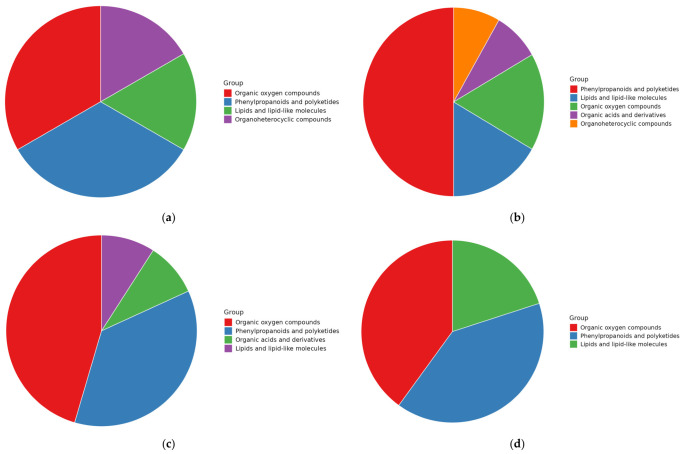
Metabolite distribution in *Eclipta prostrata* under negative ionization mode: (**a**) flower, (**b**) leaf, (**c**) root, and (**d**) stem. The inner ring represents superclasses, and the outer ring represents subclasses of the identified metabolites.

The negative ionization mode also revealed tissue-specific metabolite class distributions among the different tissues of *Eclipta prostrata*. Flower extracts were predominantly composed of organic oxygen compounds and phenylpropanoids and polyketides, followed by lipids and lipid-like molecules and organoheterocyclic compounds (Figure 11a). In contrast, leaf extracts were characterized by a higher abundance of phenylpropanoids and polyketides, together with lipids and lipid-like molecules, organic oxygen compounds, organic acids and derivatives, and organoheterocyclic compounds (Figure 11b). Root extracts were mainly composed of organic oxygen compounds and phenylpropanoids and polyketides, with smaller proportions of organic acids and derivatives and lipids and lipid-like molecules (Figure 11c). Similarly, stem extracts were dominated by phenylpropanoids and polyketides and organic oxygen compounds, while lipids and lipid-like molecules were detected at comparatively lower proportions (Figure 11d). Overall, phenylpropanoids and polyketides, together with organic oxygen compounds, represented the predominant metabolite classes detected across the different tissues of *E. prostrata* in negative ionization mode, although the relative abundance of lipids and lipid-like molecules, organic acids and derivatives, organoheterocyclic compounds, and other metabolite classes varied among plant tissues.

## 3. Discussion

In this paper, we studied the effects of different drying methods, namely freeze drying (FD), oven drying (OD), and shade drying (SD), on antioxidant properties, element presence and distribution of major chemical groups in the flowers, leaves, roots, and stems of *Eclipta prostrata*. Freeze drying generally provided superior preservation of antioxidant capacity and metabolite diversity, whereas shade drying effectively maintained selected secondary metabolite classes and mineral composition in specific tissues. In contrast, oven drying resulted in greater alterations in both phytochemical composition and metabolomic profiles. These findings indicate that no single drying method is universally optimal and that the choice of drying treatment should be determined according to the intended phytochemical or medicinal application.

### 3.1. Effect of Drying Methods on Phytochemical Preservation and Antioxidant Activity

The present study demonstrated that the effect of drying treatment on antioxidant capacity was strongly tissue-dependent; however, freeze drying generally preserved the antioxidant activity of *E. prostrata* more effectively than oven and shade drying. This trend was consistently observed across the DPPH, ABTS, and FRAP assays, particularly in flower and leaf tissues, indicating that low-temperature dehydration better maintains the antioxidant constituents responsible for free radical scavenging and ferric reducing activities. The superior antioxidant performance of freeze-dried samples is likely attributable to the absence of thermal stress, which minimizes enzymatic oxidation and degradation of heat-sensitive antioxidant metabolites during processing. In contrast, the reduced antioxidant capacity observed in oven-dried samples may be associated with thermal degradation of phenolic and flavonoid compounds, whereas the prolonged drying period during shade drying may have promoted oxidative changes that diminished radical scavenging efficiency despite retaining measurable phenolic constituents [8].

The trends observed in the present study are consistent with previous investigations of *E. prostrata*. A previous study reported DPPH IC_50_ values of 0.35 ± 0.1 and 0.95 ± 0.5 mg/mL for leaf and flower extracts prepared using a solar dryer in 99% (*v*/*v*) ethanol, together with values of 0.73 ± 0.1, 0.31 ± 0.2, and 0.49 ± 0.3 mg/mL in 70% (*v*/*v*) ethanol and 0.61 ± 0.2, 0.63 ± 0.2, and 0.65 ± 0.3 mg/mL in 30% (*v*/*v*) ethanol for stem, leaf, and flower extracts, respectively [9]. One more study discussed FRSA (%) of leaves in freeze-drying and oven-drying treatments to be 55.1 ± 2.1 and 4.9 ± 0.1, respectively [10], which was consistent with our studies, where freeze-dried leaves showed better antioxidant activity than oven-dried leaves. Comparable observations have also been reported for ABTS antioxidant activity, where oven-dried root and stem tissues exhibited reduced scavenging capacity relative to freeze-dried materials [11].

Likewise, previous FRAP studies demonstrated ferric reducing capacities of 15.877 µg/mL in shade-dried leaf methanolic extracts [12], while shade-dried whole plants showed a FRAP value of 512.23 mg AAE/g in 70% methanol extract [13].

The antioxidant responses were closely associated with the phenolic and flavonoid composition of the different plant tissues. Leaves consistently contained the highest concentrations of phenolic and flavonoid compounds, reflecting their physiological role as the primary photosynthetic organs that are continuously exposed to ultraviolet radiation and oxidative stress. Consequently, leaves accumulate greater quantities of antioxidant secondary metabolites to protect cellular structures from reactive oxygen species. Although shade drying retained comparatively high total phenolic and flavonoid contents, the corresponding antioxidant activities were not always proportionally enhanced. This discrepancy may reflect oxidation or structural modification of phenolic compounds during prolonged drying [14], whereby oxidized phenolics continue to react with the Folin–Ciocalteu reagent but exhibit reduced free radical scavenging efficiency. In contrast, the consistently lower phenolic and flavonoid contents observed following oven drying are likely attributable to thermal degradation of heat-sensitive secondary metabolites.

These findings agree with previous studies demonstrating that leaf extracts contain higher total phenolic contents than other tissues of *E. prostrata* [9]. Another study showed leaves to have a TPC value of 214.5 ± 31.7, stems 205.2 ± 47.8 and roots 198 ± 72.7 mg/g [11], while freeze drying and shade drying generally preserve phenolic compounds more effectively than oven drying because of reduced thermal degradation and oxidative damage [15].

Similarly, flavonoids such as luteolin and apigenin derivatives have been reported to accumulate predominantly in leaves, where they function as protective antioxidants against ultraviolet radiation and oxidative stress [16]. Collectively, these findings indicate that freeze drying is the most suitable method for preserving antioxidant activity, whereas shade drying may better retain certain phenolic and flavonoid constituents, highlighting the importance of selecting drying methods according to the intended phytochemical or medicinal application.

### 3.2. ICP-OES

The ICP-OES analysis demonstrated that mineral retention in *Eclipta prostrata* was influenced by both plant part and drying method, indicating that tissue-specific characteristics play an important role in determining mineral stability during dehydration. Overall, shade drying better preserved calcium and phosphorus in roots and stems, whereas oven drying was associated with higher concentrations of potassium, magnesium, sodium, and phosphorus in leaves. Flowers exhibited a mixed response, with freeze drying generally maintaining higher concentrations of several minerals. These differences may reflect variations in tissue structure, moisture removal, and the physicochemical properties of individual minerals during the drying process, which can influence their apparent concentrations on a dry-weight basis. Similar tissue- and drying-dependent variations in mineral composition have been reported in medicinal plants, suggesting that drying conditions can alter mineral retention through changes in water loss, cellular integrity, and the redistribution of soluble mineral constituents rather than mineral degradation itself. Therefore, the selection of an appropriate drying method should consider the target plant tissue and the intended nutritional or phytochemical application of the final product. Magnesium plays an important role in maintaining osmotic balance and acts as a cofactor for numerous enzymatic reactions. It also contributes to the development and proper functioning of bones and muscles as an important component of many biological reactions like DNA/RNA synthesis, energy synthesis, bone development, functioning of nerves and muscles, and maintenance of homeostasis, and it acts as a cofactor for more than 300 enzymes [17]. An appropriate K/Na ratio in food is beneficial in preventing hypertension and atherosclerosis, while calcium and phosphorus are vital for bone formation and growth [18]. Potassium is an important dietary supplement because of its role in maintaining the transmembrane electrochemical gradient, which results in proper transmission of nervous signals, contraction of muscles and kidney function [19]. The abundance of Ca, K, Mg, and P is expected due to their roles in plant growth, photosynthesis, osmotic regulation and enzymatic activity [20].

These findings were in accordance with previous studies where similar concentrations of the important macronutrients Ca, K ppm, Mg, and P were found in leaf samples using ICP-MS [21].

The concentrations of essential micronutrients were influenced by both plant part and drying method, indicating that mineral retention is tissue-dependent. Overall, roots accumulated higher concentrations of iron and copper, whereas leaves generally contained greater amounts of boron and manganese. The influence of drying treatment varied among individual elements, although shade drying generally resulted in higher concentrations of several micronutrients in roots and stems, whereas oven drying was associated with higher concentrations of selected elements, particularly iron and zinc, in flowers and leaves. These differences may be attributed to tissue-specific mineral distribution and variations in moisture removal during drying, which can influence the apparent concentration of minerals on a dry-weight basis rather than indicating actual mineral degradation or loss.

Zinc is an important dietary supplement involved in enzyme catalytic activity, protein and DNA synthesis, enhanced immune functioning, healthy pregnancy, wound healing and cellular signaling [22]. Iron, being the vital component of hemoglobin and myoglobin, has a major role in oxygen transfer to tissues and muscle metabolism, respectively. Additionally, it plays a role in better neurological development, physical growth, cell functions and synthesis of some hormones like T3 (triiodothyronine) and T4 (thyroxine) [23]. Copper acts as a cofactor for enzymes called cuproenzymes, which are involved in many important body functions like synthesis of connective tissues, energy and neurotransmitters. It also regulates gene expression, brain development and protection against the damage caused by reactive oxygen species [24]. Manganese is involved in blood clotting and hemostasis and is a cofactor for many enzymes. Manganese and iron are pharmacologically important elements with medicinal benefits; manganese exhibits antipyretic, antibiotic, antiemetic, and hypocholesterolemic activities, whereas iron is essential for the formation of hemoglobin in human blood [25].

These elevated concentrations of Fe, Zn, Cu, and Mn are important because these elements contribute to antioxidant enzyme systems and various metabolic pathways. Their presence may partially explain the antioxidant activities observed in the present study. Previous reports have shown that medicinal plants rich in micro-minerals often exhibit enhanced therapeutic and antioxidant properties due to synergistic interactions between minerals and phytochemicals [26].

The concentrations of heavy metals and non-essential trace elements, including arsenic (As), cadmium (Cd), cobalt (Co), chromium (Cr), nickel (Ni), lead (Pb), vanadium (V), aluminum (Al), and barium (Ba), varied among plant parts and drying methods, with roots generally accumulating higher concentrations than flowers, leaves, and stems. This pattern is consistent with the physiological role of roots as the primary site of metal uptake and sequestration, limiting the translocation of potentially toxic elements to aerial tissues [27]. Shade drying generally retained higher concentrations of arsenic, chromium, nickel, lead, vanadium, aluminum, and barium in roots, whereas oven drying was associated with relatively higher concentrations of chromium and vanadium in leaves and flowers. These differences are likely attributable to tissue-specific characteristics, water removal, and concentration effects during drying rather than changes in the elemental composition itself, as mineral elements are not degraded by thermal processing. Overall, these findings demonstrate that both plant part and drying method influence the measured concentrations of heavy metals and non-essential trace elements, highlighting the importance of considering drying conditions when evaluating the elemental composition and safety of medicinal plant materials.

Overall, root tissues contained the highest concentrations of arsenic, chromium, nickel, lead, and vanadium among all plant parts. Several heavy metals, including nickel and lead, were not detected in flowers and stems, while oven drying frequently resulted in non-detectable concentrations for several heavy metals in root tissues. Shade-dried roots consistently exhibited the highest concentrations of most detectable heavy metals.

The elevated metal concentrations in roots may reflect environmental exposure, soil composition, or absorption efficiency of *Eclipta prostrata*. Similar studies have reported that medicinal plant roots often accumulate higher concentrations of heavy metals than aerial parts due to prolonged interaction with contaminated soil matrices [28]. Nevertheless, most detected values remained within relatively low ranges compared to severe contamination scenarios reported in polluted environments. ICP-OES analysis revealed considerable variation in mineral concentrations among the different drying treatments, particularly for Fe in the root samples. Since all samples were dried to constant weight before grinding and analysis using the same ICP-OES protocol, these differences are unlikely to be solely attributable to sample preparation. Although no visible tissue exudates or leaching were observed during the drying process, factors associated with drying, including differences in water removal and possible redistribution of soluble mineral constituents, cannot be completely excluded. Therefore, the underlying mechanisms responsible for the observed mineral variations remain unclear and warrant further investigation.

### 3.3. Tissue-Specific Metabolomic Responses to Different Drying Methods

Multivariate metabolomic analyses were performed separately for each plant tissue in both positive and negative ionization modes to evaluate the influence of different drying methods on the metabolomic profiles of *Eclipta prostrata*. Principal component analysis (PCA) was first used to visualize global metabolic variation among freeze-dried (FD), oven-dried (OD), and shade-dried (SD) samples, followed by hierarchical clustering analysis (HCA); correlation analysis; variable importance in projection (VIP) analysis, for which a file containing the list of compounds used in the analysis has been added in the Supplementary Table S2; and chemical classification to identify tissue-specific metabolic responses and discriminative metabolite classes.

#### 3.3.1. Positive Ionization Mode

Untargeted LC–QTOF–MS metabolomic profiling identified several well-established phytochemicals previously reported in *Eclipta prostrata*, including flavonoids (quercetin, kaempferol, apigenin, luteolin, and 6-methoxyluteolin), the coumestan wedelolactone, lignan, phenolic acids (quinic acid and caffeoylquinic acid derivatives), and the benzoquinone embelin, together with sphingolipid-related metabolites such as sphinganine and phytosphingosine. These metabolites are associated with antioxidant, anti-inflammatory, hepatoprotective, and membrane-associated metabolic pathways that contribute to the medicinal properties of *E. prostrata*. Because these annotations were assigned primarily by accurate-mass matching, confirmation by MS/MS fragmentation or authentic reference standards is required for definitive metabolite identification.

Untargeted LC–QTOF–MS metabolomic profiling further demonstrated that the metabolomic response of *E. prostrata* to drying was strongly tissue-dependent. PCA, hierarchical clustering, correlation, VIP, and chemical classification analyses consistently showed that root tissues exhibited the greatest metabolomic differentiation among drying treatments, whereas flower and stem tissues remained comparatively stable and leaf tissues displayed an intermediate response. These complementary multivariate analyses indicate that drying predominantly influenced the relative abundance of specific metabolite classes rather than causing extensive alterations to the overall metabolomic composition.

Chemical classification analysis revealed that the metabolome of *E. prostrata* was dominated by phenylpropanoids and polyketides, together with lipids and lipid-like molecules, while benzenoids, organic oxygen compounds, organic acids and their derivatives, organoheterocyclic compounds, and nitrogen-containing compounds also contributed substantially to the metabolomic profiles. These metabolite classes included flavonoids, coumestans, lignans, phenolic acids, sphingolipids, fatty acyls, prenol lipids, steroid derivatives, and triterpenoid-related metabolites. This metabolite distribution agrees with previous phytochemical investigations of *E. prostrata*, which have identified flavonoids, coumestans, lignans, phenolic acids, triterpenoids, and lipid-derived metabolites as the principal bioactive constituents responsible for many of the medicinal properties of the species.

Among these metabolite classes, flavonoids and phenolic acids are recognized as major antioxidants that contribute to the free radical scavenging, anti-inflammatory, and hepatoprotective activities of *E. prostrata* [29]. Coumestans, represented by wedelolactone, together with lignans, have likewise been associated with antioxidant, antimicrobial, hepatoprotective, and cytoprotective activities [6,30]. In addition, sphingolipid-related metabolites, including sphinganine and phytosphingosine derivatives, play essential roles in membrane integrity, cellular signaling, and adaptation to dehydration stress [31,32], whereas fatty acid-derived metabolites participate in membrane remodeling during water loss. The predominance of these metabolite classes suggests that drying primarily affected metabolic pathways associated with secondary metabolism, oxidative defense, and membrane stability.

The multivariate analyses consistently identified root tissues as the most responsive to drying. PCA demonstrated the clearest separation among drying treatments in root samples, hierarchical clustering revealed distinct treatment-dependent metabolite abundance patterns, and VIP analysis identified the largest number of discriminative metabolites in this tissue. The stronger metabolic responsiveness of roots is likely related to their physiological role as storage organs, where membrane-associated lipids and secondary metabolites accumulate and are therefore more susceptible to dehydration-induced metabolic changes [33,34]. In contrast, flower and stem tissues exhibited substantial overlap among drying treatments in both PCA and hierarchical clustering analyses, indicating comparatively stable metabolomic profiles. Leaf tissues showed intermediate responses, reflecting the dynamic metabolic activity of photosynthetically active tissues that contain abundant antioxidant metabolites involved in protection against environmental and oxidative stress [35].

Correlation analysis further supported these observations by demonstrating coordinated metabolite responses within related chemical classes. In positive ionization mode, the strongest metabolite associations were primarily observed among putatively annotated sphingolipids, fatty acid derivatives, and other lipid-associated metabolites, particularly in root tissues. In contrast, negative ionization mode revealed stronger associations among flavonoids, phenolic acid derivatives, and carbohydrate-related metabolites, especially in root and stem tissues. These coordinated metabolite networks suggest that drying influences groups of metabolites belonging to related biochemical pathways rather than individual metabolites independently. Similar metabolomic responses have been reported in medicinal plants subjected to post-harvest dehydration, where membrane-associated lipids and antioxidant-related secondary metabolites are among the most responsive chemical groups because of their roles in maintaining cellular integrity during dehydration stress [31].

VIP analysis further demonstrated that different metabolite classes contributed to tissue discrimination depending on the ionization mode. In positive ionization mode, tissue differentiation was largely associated with putatively annotated sphingolipid-related metabolites, fatty acid derivatives, and the *E. prostrata*-associated lignan eudesmin, particularly in root tissues. Conversely, negative ionization mode highlighted *E. prostrata*-associated phenolic metabolites, including wedelolactone, quercetin, embelin, quinic acid, and caffeoylquinic acid derivatives, together with putatively annotated flavonoid derivatives, as the principal contributors to treatment discrimination. These findings indicate that positive and negative ionization modes provided complementary information, with positive mode preferentially detecting lipid-associated metabolites and negative mode better resolving phenolic compounds and related secondary metabolites.

Collectively, these findings demonstrate that drying influences the metabolomic profile of *E. prostrata* in a tissue-specific manner. Rather than causing extensive changes in overall metabolite composition, drying primarily altered the relative abundance of bioactive metabolite classes. The magnitude of these changes varied among plant tissues, with root tissues exhibiting the greatest metabolic responsiveness and flower and stem tissues remaining comparatively stable. These observations highlight the importance of selecting an appropriate drying strategy according to the target plant tissue and the desired preservation of pharmacologically important metabolites in *E. prostrata*.

#### 3.3.2. Negative Ionization Mode

Untargeted LC–QTOF–MS metabolomic profiling in negative ionization mode demonstrated that the influence of drying treatment on *Eclipta prostrata* was strongly tissue-dependent. Although PCA and PERMANOVA indicated that drying treatments did not produce statistically significant global alterations in metabolite composition, hierarchical clustering, correlation, VIP, and chemical classification analyses consistently revealed tissue-specific differences in the relative abundance of selected metabolite classes. These observations suggest that drying primarily modulated metabolic regulation within existing biochemical pathways rather than inducing extensive metabolomic reorganization, particularly considering the limited number of biological replicates included in the present study.

Among the four tissues, flower and stem tissues exhibited comparatively stable metabolomic profiles across the drying treatments. The substantial overlap among treatments observed in the PCA score plots, together with the absence of distinct clustering patterns in the hierarchical clustering analysis, indicates that the metabolite composition of these aerial tissues was only moderately affected by dehydration. Flowers represent terminal reproductive structures that undergo relatively short developmental periods, whereas stems primarily function in mechanical support and vascular transport through lignified tissues [36]. Consequently, coordinated metabolic adjustments during drying may be less pronounced than those occurring in metabolically active storage tissues, resulting in relatively modest treatment-dependent alterations in the abundance of individual metabolites.

In contrast, root tissues exhibited the strongest metabolomic response to drying. The greater treatment-associated separation observed in PCA, together with distinct metabolite clustering patterns in hierarchical clustering analysis and coordinated metabolite associations revealed by correlation analysis, indicate that roots were the most responsive tissue to dehydration. This enhanced responsiveness is likely associated with the physiological role of roots as storage organs that accumulate secondary metabolites, carbohydrates, and membrane-associated compounds. Such metabolites are known to participate in osmotic adjustment, oxidative defense, and membrane stabilization during dehydration, making root tissues particularly susceptible to changes in metabolite abundance under different drying conditions [33,34]. Leaf tissues displayed intermediate responses, reflecting their active metabolic functions in photosynthesis and antioxidant metabolism, where dehydration influences both primary and secondary metabolic pathways involved in protection against oxidative stress [35].

Correlation analysis further demonstrated coordinated regulation among metabolites belonging to related biochemical pathways. In negative ionization mode, the strongest positive metabolite associations were primarily observed among flavonoid-related metabolites, phenolic acid derivatives, and carbohydrate-associated metabolites, particularly in root and stem tissues. These coordinated metabolite networks suggest that dehydration influenced interconnected metabolic pathways rather than individual metabolites independently. Similar coordinated responses have been reported in medicinal plants subjected to post-harvest drying, where antioxidant-related secondary metabolites are regulated simultaneously to maintain cellular homeostasis and minimize oxidative damage during water loss [31].

Chemical classification analysis further demonstrated that the negative-ionization metabolome of *E. prostrata* was dominated by phenylpropanoids and polyketides together with organic oxygen compounds, while lipids and lipid-like molecules, organic acids and their derivatives, organoheterocyclic compounds, benzenoids, and nitrogen-containing compounds also contributed to the metabolomic profiles of different tissues. Leaves were relatively enriched in flavonoid-derived metabolites, whereas roots contained a broader distribution of phenolic acids, carbohydrates, and lipid-associated metabolites. This distribution is consistent with previous phytochemical investigations demonstrating that *E. prostrata* contains abundant flavonoids, coumestans, lignans, phenolic acids, and triterpenoid-related metabolites that collectively contribute to its medicinal properties [6,32].

Among these metabolite classes, coumestans, particularly wedelolactone, represent characteristic constituents of *E. prostrata* and have been widely associated with antioxidant, anti-inflammatory, hepatoprotective, antimicrobial, and anticancer activities [37,38]. Likewise, flavonoids, including quercetin, together with luteolin- and apigenin-related metabolites, contribute substantially to the antioxidant capacity of the species through reactive oxygen species scavenging and protection against oxidative stress. Phenolic acids, represented by quinic acid and caffeoylquinic acid derivatives, further contribute to antioxidant defense and phenylpropanoid metabolism. The detection of oxylipin-related metabolites, including 10S-HODE, also suggests modulation of lipid-mediated signaling pathways during dehydration, as oxylipins are known to regulate stress responses and membrane remodeling under environmental stress conditions. In addition, the occurrence of triterpenoid- and saponin-related metabolites agrees with previous reports describing their hepatoprotective, anti-inflammatory, and immunomodulatory activities in *E. prostrata* [5].

VIP analysis further supported these observations by identifying tissue-specific discriminative metabolites associated with the drying treatments. In leaf tissues, discrimination was primarily associated with *E. prostrata*-related flavonoids and phenolic acids, including wedelolactone, together with putatively annotated flavonoid sulfates and carbohydrate-related metabolites. Root tissues were characterized by quercetin, wedelolactone, and quinic acid, together with putatively annotated flavonoid glycosides and carbohydrate-derived metabolites, whereas stem tissues were mainly differentiated by wedelolactone, embelin, quercetin, and putatively annotated flavonoid derivatives. These findings indicate that drying predominantly influenced the relative abundance of bioactive phenolic metabolites and their associated metabolic pathways rather than inducing qualitative changes in metabolite composition.

Collectively, the multivariate analyses demonstrate that the metabolomic response of *E. prostrata* to drying is highly tissue-dependent. Root tissues consistently exhibited the greatest metabolic responsiveness, supported by clearer treatment-associated separation in PCA, more distinct hierarchical clustering patterns, stronger metabolite correlation networks, and a greater number of discriminative metabolites identified by VIP analysis. Leaf tissues displayed intermediate responses, whereas flower and stem tissues remained comparatively stable despite moderate variations in the abundance of individual metabolites. These findings indicate that drying primarily affects the regulation and preservation of bioactive metabolite classes rather than fundamentally altering the overall metabolomic composition of *E. prostrata*, highlighting the importance of considering tissue type when selecting post-harvest drying strategies for medicinal plant materials.

## 4. Materials and Methods

### 4.1. Plant Materials and Drying

Plant material was obtained from Nakhon Pathom city, Nakhon Pathom province, Thailand. The plant material was thoroughly washed with water and patted dry. Afterwards, it was separated into four parts, i.e., flowers, leaves, roots and stems. The separated parts were then dried using three different drying methods, which were freeze drying, oven drying and shade drying. For freeze drying, the plant material was lyophilized (VirTis Genesis 25L, Sithiporn Associates, Bangkok, Thailand) at −60 °C, 100 m Torr, for 21 h. For oven drying, the plant material was placed in a hot air oven (Ethos Up, Milestones, Sorisole, Italy) at 60 °C for 20–24 h. Lastly, for shade drying, the plant material was placed in a well-ventilated room, away from direct sunlight, at approximately 22–30 °C temperature, until the plant was fully dried. Following the drying methods, the plant parts were ground to powder using a pestle and mortar, which were stored in a −20 °C freezer.

### 4.2. Extraction

Maceration of the sample was performed by soaking the different parts in absolute ethanol at a ratio of 1:30 (*w*/*v*) and shaking at 160 rpm for 24 h at room temperature (25 ± 5 °C). All extracts were filtered through Whatman No. 1 paper (Whatman, OH, Cytiva, Marlborough, MA, USA) followed by rotary evaporation. All the evaporated samples were dissolved in 10 mL of absolute ethanol to obtain crude extract, which was then stored at −20 °C until further use.

### 4.3. DPPH Radical Scavenging Activity

The free radical scavenging activity was evaluated using the DPPH assay with minor modifications [39]. 1,1-diphenyl-2-picrylhydrazyl (DPPH) assay was used to assess the antioxidant potential of the plant sample with minor adjustments. Ascorbic acid was used as the standard. The stock solution of DPPH was prepared using 24 mg of DPPH in 100 mL methanol, which was then diluted using 10 mL stock in 45 mL of methanol. The absorbance of the working solution was adjusted to 0.9 at 517 nm. Afterwards, 100 µL of working DPPH solution was mixed with 100 µL of plant extracts, which had concentrations starting at 0.0078125 to 1.000 mg/mL, in a 96-well plate. After incubating in the dark for 30 min, absorbance was measured at 517 nm using a microplate reader (CLARIOstar Plus, Ortenberg, Germany). The free radical scavenging activity was calculated by (Equation (1)):

% Inhibition = (A_control_ − A_sample_)/A_control_ × 100. (1)

The IC_50_ values were calculated from the inhibition curves and expressed as mg/mL of extract.

### 4.4. ABTS Radical Scavenging Activity

ABTS assay was performed using [39] to evaluate the antioxidant potential of 2,2′-azio-bis(3-ethylbenzothiazoline-6-sulfonic acid) diammonium salt (ABTS). Trolox was used as the standard. ABTS cations were generated by initiating a reaction between 19.5 mg of ABTS and 3.3 mg of potassium persulfate (K_2_S_2_O_8_) and dissolving it in water. The colorless ABTS solution turns dark blue-green after incubation in the dark for 12–16 h at room temperature. Subsequently, the ABTS solution is diluted using 80% ethanol to obtain a 1% solution. The absorbance of the solution is adjusted to 0.7 ± 0.02 at 734 nm. A 190 µL measure of this solution is added to a 96-well plate along with 10 µL of sample, ranging in concentration from 0.0078125 to 1.000 mg/mL, which was incubated for 10 min, and the absorbance was measured at 734 nm. The % radical scavenging was calculated using (Equation (1)):

% Inhibition = (A_control_ − A_sample_)/A_control_ × 100. (1)

The IC_50_ values were calculated from the dose–response curves and expressed as mg/mL of extract.

### 4.5. Ferric Reducing Antioxidant Power (FRAP) Assay

FRAP was determined following the adjustments in [39]. Initially, 10 mM tripyridyltriazine (TPTZ) was prepared by dissolving 31.2 mg of TPTZ in 10 mL of 40 mM HCl. A 300 mM acetate buffer (pH 3.6) solution was prepared, which was used along with previously made TPTZ and a 20 mM Iron III chloride hexahydrate (FeCl_3_.6H_2_O) solution at a ratio of 10:1:1 to make the FRAP reagent. The FRAP solution appeared pale yellow after combining the reagents. After adding 10 µL of sample solution of concentrations 0.0078125 to 1.000 mg/mL into a 96-well plate, 190 µL of the freshly prepared FRAP reagent was added. This was followed by incubation in the dark at room temperature for 30 min. Absorbance was measured at 593 nm thereafter. The FRAP values were calculated using an ascorbic acid standard curve (0–1000 µg/mL), and the results were expressed as milligrams of ascorbic acid equivalent per mg AAE/g extract using the following formula:

FRAP (mgAAE/g) = (C_AA × V × DF × 100)/sample weight (g)(2)

where C_AA = concentration of ascorbic acid from the standard curve (mg/mL), V = volume of sample solution (mL) and DF = dilution factor.

### 4.6. Total Phenolic Content

Total phenolic content of the plant parts was determined through the modified Folin–Ciocalteu method [40]. For this experiment, gallic acid (purity ≥ 98%, Fluka, Buchs, Switzerland) was used as the standard at concentrations of 0.02, 0.04, 0.08, 0.16, 0.32 and 0.64 mg/mL, which were prepared using 80% methanol. Each gallic acid standard solution and plant extract sample was reacted with Folin–Ciocalteu reagent (Merck, Darmstadt, Germany) under identical conditions. After 6 min, 125 µL of 7% sodium carbonate (Na_2_CO_3_) solution and 100 µL of distilled water were added. The reaction mixture was incubated for 90 min, and the absorbance was measured at 760 nm using a microplate reader. The total phenolic content was calculated from the gallic acid calibration curve and is expressed as milligrams of gallic acid equivalents per gram of dry sample (mg GAE/g sample).

### 4.7. Total Flavonoid Content

The measurement of total flavonoid content was done by mixing 25 µL of the sample with 75 µL of 7% NaNO_2_ solution and 12.5 µL of distilled water using the modified method [41]. After 5 min, 15 µL of 10% AlCl_3_ was added to the mixture. After standing for 5 min, 50 µL of 1 M NaOH and 27.5 µL of distilled water were added and allowed to sit for another 5 min. Lastly, absorbance was measured at 510 nm, and the results are expressed in mg of quercetin per gram of dry sample (mg QE/ g sample).

### 4.8. Inductively Coupled Plasma Optical Emission Spectroscopy (ICP-OES) Analysis

Elemental analysis of the *Eclipta prostrata* was performed using an inductively coupled plasma-optical emission spectrometer (ICP-OES, Agilent 5800, Santa Clara, CA, USA). Approximately 0.5 g of dried powdered sample from each group was weighed into Teflon digestion vessels. Each sample was digested with 10 mL concentrated nitric acid (HNO_3_) and 1 mL hydrogen peroxide (H_2_O_2_) (10:1, *v*/*v*) (sample-to-digestion solution ratio = 0.5 g:11 mL) using a microwave digestion system (Milestone Ethos Up, Sorisole, Italy). The digestion program ramped the temperature to 190 °C over 25 min and maintained it at 190 °C for 20 min according to the Methods Library for the SK-15 ET high-pressure rotor. After cooling, the digested solutions were filtered through a 0.45 μm, 13 mm nylon syringe filter and quantitatively transferred to 25 mL volumetric flasks, then diluted to volume with deionized water prior to ICP-OES analysis.

The concentrations of Ca (393.366 nm), K (766.491 nm), Mg (280.270 nm), Na (588.995 nm), P (213.618 nm), B (249.772 nm), Cu (324.754 nm), Fe (238.204 nm), Mn (257.610 nm), Zn (213.857 nm), Al (396.152 nm), Ba (455.403 nm), Si (251.611 nm), As (188.980 nm), Cd (226.502 nm), Co (238.892 nm), Cr (205.560 nm), Ni (216.555 nm), Pb (220.353 nm), and V (311.837 nm) were determined using the Agilent 5800 ICP-OES. Mineral concentrations were expressed as ppm (mg/kg) on a dry-weight basis (DW).

### 4.9. Liquid Chromatography–Quadrupole Time-of-Flight Mass Spectrometry (LC-QTOF-MS) Characterization

LC-QTOF-MS analysis was performed using a modified method [42] with an Agilent 6546 Q-TOF mass spectrometer (QTOF-MS, Santa Clara, CA, USA) equipped with a Dual AJS ESI source operating in positive and negative ion modes. Chromatographic separation was performed on an Agilent ZORBAX Eclipse Plus C18 Rapid Resolution HD column (2.1 × 150 mm, 1.8 µm; Agilent Technologies, Santa Clara, CA, USA) maintained at 40 °C. The mobile phases consisted of water containing 0.1% formic acid (A) and acetonitrile (B) at a flow rate of 0.2 mL min^−1^. The gradient program was as follows: 30% B as the initial condition, linearly increased to 100% B over 15 min, followed by a 2 min hold at 100% B, giving a total run time of 17 min. The injection volume was 1 µL. Mass spectra were acquired over an *m*/*z* range of 100–1700 with a scan rate of 1 spectrum/s. The capillary voltage was set to 3500 V, fragmentor voltage to 175 V, gas temperature to 320 °C, sheath gas temperature to 350 °C, and nebulizer pressure to 35 psig, and the injection volume was 1 µL. The results from both positive and negative modes were analyzed using statistical analysis in MetaboAnalyst 6.0. All metabolite identifications were putative unless confirmed by authentic standards or comprehensive MS/MS validation. Consequently, only metabolites with strong literature support and direct relevance to the objectives of this study were discussed in detail, while other tentative annotations were not further interpreted.

### 4.10. Statistical Analysis

All experiments were performed in triplicate, and the results are presented as the mean ± standard deviation (SD). Statistical analyses of antioxidant activity, total phenolic content, total flavonoid content, and elemental composition were performed using IBM SPSS Statistics Version 25 (IBM Corp., Armonk, NY, USA). Differences among drying treatments were evaluated by one-way analysis of variance (ANOVA), followed by Tukey’s honestly significant difference (HSD) post hoc test. A value of *p* < 0.05 was considered statistically significant.

Untargeted metabolomic data were processed using Metaboanalyst 6.0. Principal component analysis (PCA), hierarchical clustering analysis (HCA), variable importance in projection (VIP) analysis, correlation analysis, and chemical classification were performed to evaluate tissue-specific metabolic differences among drying treatments. Pairwise differences in metabolomic profiles were assessed using PERMANOVA with 999 permutations, based on Pearson’s correlation (*r*) similarity; *p*-values were adjusted for multiple comparisons using the Benjamini–Hochberg false discovery rate (FDR < 0.05) and a correlation cutoff of 0.7 was chosen.

## 5. Conclusions

In this study, antioxidant assays, ICP-OES elemental profiling, and LC-QTOF-MS metabolomics analyses demonstrated that drying methods had a notable impact on the phytochemical composition and antioxidant potential of different parts of *Eclipta prostrata*. Freeze drying generally exhibited enhanced scavenging activities, producing the lowest DPPH and ABTS IC_50_ values and the highest FRAP values, especially in flower and leaf samples. TPC and TFC were highest in shade-dried leaves, indicating that mild drying conditions favor flavonoid and phenolic compound retention, although these compounds may not always translate directly into stronger radical scavenging activities, as seen in the scavenging assay results. ICP-OES analysis revealed the presence of nutritionally important minerals, including Ca, K, Mg, Fe, Zn, and P, with relatively higher concentrations generally observed in the stem and root tissues under shade-drying conditions. From a safety perspective, most heavy metals were detected at low concentrations across the different plant parts and drying treatments. However, the shade-dried root samples exhibited elevated concentrations of arsenic and chromium, exceeding the recommended permissible limits and indicating a potential toxicological concern. These findings suggest that, in addition to preserving phytochemical composition, the selection of appropriate plant parts and drying methods should also consider mineral composition and heavy metal accumulation to ensure the safety and quality of *Eclipta prostrata* for medicinal and nutraceutical applications. Untargeted LC-QTOF-MS metabolomics further revealed tissue-specific responses to drying treatments, with roots being the tissue most influenced by drying conditions, giving the greatest variations in metabolic profile, while stems were the least affected. Drying primarily affected lipids and their derivatives, flavonoids, anthraquinones, alkaloids, and other secondary metabolites involved in antioxidant defense and stress adaptation. Across all samples, freeze-dried plant parts consistently preserved a broad range of bioactive compounds, followed by shade-dried plant parts, while oven-dried samples exhibited the lowest retention. Overall, freeze drying appears to be the most suitable drying method for maximizing the antioxidant potential and metabolic integrity in *E. prostrata*, supporting its potential use in pharmaceutical, nutraceutical, and functional food applications. Although shade-dried leaves showed the highest TPC and TFC values, freeze-dried samples showed a consistent preservation of better antioxidant activity and metabolic diversity across all four parts of the plant. Future research should focus on validating the key metabolites identified in this study while investigating how drying-induced metabolomic changes affect the biological activities of the plant.

## Figures and Tables

**Table 1:** Antioxidant activity of parts of *Eclipta prostrata* following different drying methods.

Drying Method	Parts of Plant	Free Radical Scavenging Assay		
		DPPH Radical Scavenging Assay (mg/mL)	ABTS Radical Scavenging Assay IC_50_ (mg/mL)	Ferric Reducing Antioxidant Power (FRAP) Assay (mg AAE/g)
Freeze dry	Flower	0.11 ± 0.01 ^b^	1.19 ± 0.17 ^b^	55.91 ± 6.23 ^a^
	Leaves	0.31 ± 0.01 ^b^	1.44 ± 0.25 ^c^	33.71 ± 1.87 ^a^
	Root	0.43 ± 0.19 ^c^	1.88 ± 0.37 ^b^	32.78 ± 7.49 ^a^
	Stem	0.38 ± 0.02 ^b^	1.95 ± 0.65 ^b^	30.54 ± 2.63 ^a^
Oven dry	Flower	0.98 ± 0.06 ^a^	3.31 ± 0.23 ^a^	17.56 ± 0.05 ^b^
	Leaves	0.56 ± 0.04 ^b^	2.07 ± 0.06 ^b^	16.98 ± 0.38 ^b^
	Root	1.56 ± 0.05 ^a^	15.12 ± 2.12 ^a^	11.30 ± 0.06 ^b^
	Stem	0.65 ± 0.13 ^a^	4.23 ± 0.10 ^a^	14.90 ± 0.14 ^b^
Shade dry	Flower	0.95 ± 0.01 ^a^	3.25 ± 0.32 ^a^	17.34 ± 0.27 ^b^
	Leaves	1.9 ± 0.52 ^a^	3.93 ± 0.21 ^a^	12.34 ± 0.26
	Root	0.78 ± 0.02 ^b^	3.22 ± 0.52 ^b^	16.91 ± 0.93 ^b^
	Stem	0.81 ± 0.01 ^a^	3.52 ± 0.41 ^a^	16.53 ± 0.33 ^b^

Values are expressed as mean ± standard deviation (*n* = 3). Different lowercase superscript letters within the same plant part and within the same antioxidant assay indicate significant differences among drying methods according to Tukey’s HSD test (*p* < 0.05).

**Table 2:** Macronutrient composition of *Eclipta prostrata* parts following different drying methods.

Macro-Minerals						
Plant Part	Drying Method	Ca	K	Mg	Na	P
Flower	Freeze	9664.43 ± 8.80 ^a^	2732.49 ± 19.68 ^b^	3384.36 ± 22.67 ^a^	2162.75 ± 11.05 ^a^	7374.45 ± 14.39 ^a^
	Oven	6093.96 ± 5.54 ^b^	2694.92 ± 24.06 ^b^	3287.52 ± 14.10 ^b^	2127.15 ± 18.45 ^b^	7054.26 ± 23.90 ^b^
	Shade	1139.79 ± 2.45 ^c^	2956.85 ± 7.59 ^a^	3216.49 ± 8.40 ^c^	2146.58 ± 10.22 ^ab^	6649.91 ± 10.51 ^c^
Leaf	Freeze	2014.58 ± 4.26 ^b^	3029.57 ± 11.04 ^b^	4374.86 ± 15.81 ^c^	7333.44 ± 34.39 ^b^	5855.26 ± 74.45 ^b^
	Oven	1988.67 ± 3.51 ^c^	3153.26 ± 39.01 ^a^	4632.13 ± 42.94 ^a^	7866.19 ± 54.75 ^a^	6241.18 ± 51.15 ^a^
	Shade	2029.96 ± 4.27 ^a^	2856.72 ± 14.14 ^c^	4458.39 ± 22.60 ^b^	7223.09 ± 42.70 ^b^	5792.75 ± 48.64 ^b^
Root	Freeze	6416.11 ± 7.20 ^b^	2427.12 ± 14.65 ^b^	1328.13 ± 5.97 ^b^	2406.58 ± 9.52 ^b^	1642.26 ± 10.09 ^b^
	Oven	216.46 ± 0.44 ^c^	66.10 ± 6.61 ^c^	50.51 ± 6.85 ^c^	95.35 ± 10.42 ^c^	61.37 ± 8.60 ^c^
	Shade	6577.18 ± 4.90 ^a^	2996.54 ± 10.78 ^a^	1545.39 ± 12.92 ^a^	2864.58 ± 14.38 ^a^	2086.07 ± 10.04 ^a^
Stem	Freeze	11,033.56 ± 13.46 ^b^	3780.16 ± 16.70 ^a^	2719.79 ± 14.33 ^a^	4450.49 ± 11.41 ^a^	4140.32 ± 26.91 ^a^
	Oven	1221.91 ± 3.15 ^c^	3464.61 ± 125.24 ^b^	2333.82 ± 92.44 ^c^	4309.09 ± 174.17 ^a^	3517.41 ± 173.19 ^b^
	Shade	11,892.62 ± 7.44 ^a^	3659.62 ± 17.31 ^ab^	2567.56 ± 16.58 ^b^	3769.75 ± 19.04 ^b^	4093.61 ± 57.91 ^a^

Values expressed in ppm. Values are expressed as mean ± standard deviation (*n* = 3). Different superscript letters within the same plant part and element indicate significant differences among drying methods according to Tukey’s HSD test (*p* < 0.05).

**Table 3:** Trace mineral composition of *Eclipta prostrata* parts following different drying methods.

Trace Minerals							
Plant Part	Drying Method	B	Cu	Fe	Mn	Zn	Si
Flower	Freeze	36.93 ± 0.11 ^b^	11.27 ± 0.07 ^a^	190.04 ± 0.11 ^b^	117.94 ± 0.32 ^a^	35.75 ± 0.18 ^b^	147.80 ± 0.63 ^b^
	Oven	37.6 ± 0.20 ^a^	11.45 ± 0.05 ^a^	277.56 ± 1.83 ^a^	107.44 ± 0.90 ^b^	37.06 ± 0.31 ^a^	140.98 ± 0.41 ^c^
	Shade	36.76 ± 0.17 ^b^	11.26 ± 0.15 ^a^	154.71 ± 0.87 ^c^	119.18 ± 0.24 ^a^	32.01 ± 0.18 ^c^	270.73 ± 2.10 ^a^
Leaf	Freeze	70.16 ± 0.09 ^c^	14.01 ± 0.03 ^c^	269.11 ± 3.46 ^c^	253.65 ± 0.43 ^ab^	28.99 ± 0.08 ^c^	97.00 ± 0.53 ^c^
	Oven	78.19 ± 0.89 ^b^	15.82 ± 0.19 ^a^	451.20 ± 5.65 ^a^	252.81 ± 2.35 ^b^	32.96 ± 0.35 ^a^	137.95 ± 1.67 ^b^
	Shade	80.29 ± 0.58 ^a^	15.18 ± 0.06 ^b^	412.30 ± 1.11 ^b^	271.18 ± 11.90 ^a^	30.67 ± 0.29 ^b^	163.72 ± 0.79 ^a^
Root	Freeze	21.11 ± 0.08 ^b^	21.68 ± 0.10 ^b^	1201.67 ± 5.82 ^b^	76.49 ± 0.29 ^b^	21.41 ± 0.11 ^b^	280.41 ± 1.94 ^a^
	Oven	0.15 ± 0.08 ^c^	ND	2.71 ± 0.54 ^c^	2.24 ± 0.35 ^c^	0.20 ± 0.09 ^c^	1.68 ± 0.22 ^c^
	Shade	29.03 ± 0.12 ^a^	35.50 ± 0.06 ^a^	2383.57 ± 13.09 ^a^	106.61 ± 0.41 ^a^	28.77 ± 0.21 ^a^	116.59 ± 0.72 ^b^
Stem	Freeze	28.68 ± 0.10 ^b^	14.54 ± 0.03 ^b^	141.49 ± 1.14 ^b^	114.37 ± 0.67 ^b^	24.69 ± 0.11 ^b^	166.03 ± 0.88 ^a^
	Oven	26.36 ± 1.14 ^c^	12.64 ± 0.51 ^c^	129.61 ± 4.96 ^c^	120.82 ± 5.88 ^b^	18.90 ± 0.91 ^c^	167.53 ± 6.39 ^a^
	Shade	32.46 ± 0.23 ^a^	17.77 ± 0.07 ^a^	180.52 ± 3.27 ^a^	130.72 ± 0.81 ^a^	26.96 ± 0.21 ^a^	163.29 ± 1.35 ^a^

Values expressed in ppm. (ND = Not detected). Values are expressed as mean ± standard deviation (*n* = 3). Different superscript letters within the same plant part and element indicate significant differences among drying methods according to Tukey’s HSD test (*p* < 0.05).

**Table 4:** Heavy metal composition of *Eclipta prostrata* parts following different drying methods.

Heavy Metals										
Plant Part	Drying Method	As	Cd	Co	Cr	Ni	Pb	Al	Ba	V
Flower	Freeze	1.15 ± 0.20 ^a^	ND	0.04 ± 0.06 ^a^	ND	ND	ND	293.49 ± 1.05 ^b^	6.39 ± 0.02 ^c^	ND
	Oven	0.83 ± 1.09 ^a^	0.01 ± 0.02 ^b^	0.03 ± 0.05 ^a^	2.10 ± 0.05 ^a^	ND	ND	460.46 ± 2.81 ^a^	6.97 ± 0.05 ^b^	0.047 ± 0.04 ^a^
	Shade	0.77 ± 0.43 ^a^	ND	0.12 ± 0.03 ^a^	ND	ND	ND	254.08 ± 1.14 ^c^	8.06 ± 0.03 ^a^	ND
Leaf	Freeze	0.76 ± 0.25 ^a^	0.01 ± 0.02 ^b^	0.03 ± 0.09 ^a^	0.11 ± 0.03 ^c^	ND	ND	492.55 ± 2.14 ^c^	14.31 ± 0.02 ^b^	0.18 ± 0.05 ^c^
	Oven	1.53 ± 0.78 ^a^	0.05 ± 0.03 ^ab^	0.05 ± 0.07 ^a^	1.15 ± 0.04 ^a^	0.01 ± 0.08 ^a^	ND	883.68 ± 9.32 ^a^	17.16 ± 0.20 ^a^	0.58 ± 0.05 ^a^
	Shade	0.89 ± 0.43 ^a^	ND	0.043 ± 0.11 ^a^	0.400 ± 0.01 ^b^	0.02 ± 0.09 ^a^	ND	766.93 ± 3.97 ^b^	17.24 ± 0.05 ^a^	0.41 ± 0.05 ^b^
Root	Freeze	2.09 ± 0.62 ^b^	0.04 ± 0.02 ^a^	0.20 ± 0.18 ^a^	1.78 ± 0.07 ^b^	0.31 ± 0.01 ^b^	0.49 ± 0.21 ^b^	1147.24 ± 4.12 ^b^	20.53 ± 0.34 ^b^	1.430 ± 0.0 ^b^
	Oven	0.43 ± 0.15 ^c^	ND	ND	ND	ND	ND	3.49 ± 0.76 ^c^	0.14 ± 0.05 ^c^	ND
	Shade	6.44 ± 0.88 ^a^	0.14 ± 0.00 ^a^	0.26 ± 0.07 ^a^	10.01 ± 0.10 ^a^	1.36 ± 0.13 ^a^	1.56 ± 0.27 ^a^	2819.20 ± 13.74 ^a^	37.11 ± 0.22 ^a^	3.90 ± 0.07 ^a^
Stem	Freeze	0.04 ± 0.30 ^a^	0.02 ± 0.03 ^a^	0.02 ± 0.01 ^b^	ND	ND	ND	232.07 ± 1.52 ^b^	15.23 ± 0.03 ^b^	ND
	Oven	1.01 ± 0.18 ^a^	ND	0.04 ± 0.03 ^b^	ND	ND	ND	204.23 ± 7.80 ^c^	17.43 ± 0.71 ^a^	ND
	Shade	1.18 ± 0.77 ^a^	0.08 ± 0.01 ^a^	0.15 ± 0.032 ^a^	1.76 ± 0.03 ^a^	ND	ND	291.54 ± 1.83 ^a^	17.08 ± 0.08 ^a^	ND

Values expressed in ppm. (ND = Not detected). Values are expressed as mean ± standard deviation (*n* = 3). Different superscript letters within the same plant part and element indicate significant differences among drying methods according to Tukey’s HSD test (*p* < 0.05).

## Data Availability

The original contributions presented in this study are included in the article; however, further queries can be directed to the corresponding author.

## Supplementary Materials

The following supporting information can be downloaded at https://www.mdpi.com/article/10.3390/ijms27157014/s1.

## Abbreviations

The following abbreviations are used in this manuscript:

ABTS	2,2′-Azino-bis (3-ethylbenzothiazoline-6-sulfonic acid)
AAE	Ascorbic Acid Equivalent
DPPH	2,2-Diphenyl-1-picrylhydrazyl
FD	Freeze Drying
FRAP	Ferric Reducing Antioxidant Power
GAE	Gallic Acid Equivalent
ICP-OES	Inductively Coupled Plasma–Optical Emission Spectrometry
LC-QTOF-MS	Liquid Chromatography–Quadrupole Time-of-Flight Mass Spectrometry
ND	Not detected
OD	Oven Drying
PCA	Principal Component Analysis
PERMANOVA	Permutational Multivariate Analysis of Variance
PLS-DA	Partial Least Squares Discriminant Analysis
QE	Quercetin Equivalent
ROS	Reactive Oxygen Species
SD	Shade Drying
TFC	Total Flavonoid Content
TPC	Total Phenolic Content
VIP	Variable Importance in Projection
WHO	World Health Organization
EFSA	European Food Safety Authority
PR	Population Reference Intake
AI	Adequate Intake
