# Supplementary material for: Tissue-Specific Metabolite Profiling and Antioxidant Potential of Eclipta prostrata Following Different Drying Treatments
Source: Int J Mol Sci. 2026 Aug 4;27(15):7014. doi: 10.3390/ijms27157014 (PMC13467206; doi:10.3390/ijms27157014)
Supplement: Supplementary file 1 [file ijms-27-07014-s001.zip › ijms-4448762-supplementary.pdf]

Table S1. Effects of drying methods on the total phenolic and flavonoid content in different parts of *Eclipta prostrata.*

Bioactive components	Plant Part	Freeze Drying	Shade Drying	Oven Drying
TPC (mg GAE/g sample)	Flower	18.63 ± 0.52[a]	5.69 ± 0.76[b]	5.49± 0.39[b]
	Leaf	14.20 ±1.77[b]	43.30 ± 0.53[a]	6.94± 0.69[c]
	Root	6.80 ± 0.20[a]	4.38 ± 0.07[b]	2.81 ± 0.06[c]
	Stem	15.10 ± 1.06[a]	3.55± 0.40[b]	3.08± 0.09[b]
**Bioactive components**	**Plant Part**	**Freeze Drying**	**Shade Drying**	**Oven Drying**
TFC (mg QE/ g sample)	Flower	12.36± 0.59[a]	10.86± 0.17[b]	11.78± 1.23[a]
	Leaf	14.88±0.42[b]	17.63± 0.55[a]	9.40± 0.71[c]
	Root	6.18± 0.08[a]	5.40± 0.70[a]	3.46± 0.25[b]
	Stem	11.78± 0.52[a]	5.28± 0.50[b]	5.70± 0.82[b]

* Values are expressed as mean ± standard deviation (n = 3).

* Different superscript letters within the same row indicate significant differences according to Tukey's HSD test at p<0.05p.

Table S2. Major abundant metabolites identified across different drying methods in *Eclipta prostrata* tissues.

Sample	Condition	Name	Mass	Formula	m/z	RT	Ion Mode	Score
Stem	Freeze dry	Nigerose	342.1156	C12 H22 O11	341.1083	1.499	-	97.26
		5Z-Caffeoylquinic acid	354.0942	C16 H18 O9	353.0869	2.723	-	95.95
		Quercetin	302.042	C15 H10 O7	301.0347	2.201	-	94.92
		C17 Sphinganine	287.2822	C17 H37 N O2	288.2895	7.365	+	99.54
		C16 Sphinganine	273.2668	C16 H35 N O2	274.274	6.554	+	99.96
		Phytosphingosine	317.2928	C18 H39 N O3	318.3001	6.669	+	99.81
	Oven dry	Kaempferol 8-C-sulfate	366.0035	C15 H10 O9 S	364.9963	2.903	-	95.87
		Embelin	294.1825	C17 H26 O4	293.1752	8.545	-	95.88
		Embelin	294.1825	C17 H26 O4	293.1752	8.545	-	95.88
		Phytosphingosine	317.2928	C18 H39 N O3	318.3001	6.669	+	99.81
		Nigerose	342.1157	C12 H22 O11	365.105	1.513	+	98.76
		9-Heneicosene	294.3286	C21 H42	312.3625	12.27	+	99.71
	Shade dry	Nigerose	342.1156	C12 H22 O11	341.1083	1.499	-	97.26
		Heudelottin C	698.3671	C39 H54 O11	697.36	11.874	-	89.59
		Quercetin	302.042	C15 H10 O7	301.0347	2.201	-	94.92
		Gravolenic acid	280.0941	C14 H16 O6	281.1014	8.281	+	98
		9-Tricosene	322.3592	C23 H46	340.393	13.644	+	95.87
		9-Heneicosene	294.3286	C21 H42	312.3625	12.27	+	99.71

Flower	Freeze dry	Quinic acid	354.0942	C16 H18 O9	353.0869	2.723	-	95.95
		Nigerose	342.1156	C12 H22 O11	341.1083	1.499	-	97.26
		DL-2-hydroxy stearic acid	300.2656	C18 H36 O3	299.2583	13.793	-	93.67
		C17 Sphinganine	287.2822	C17 H37 N O2	288.2895	7.365	+	99.54
		C16 Sphinganine	273.2668	C16 H35 N O2	274.274	6.554	+	99.96
		Sphinganine	273.2668	C16 H35 N O2	274.274	6.554	+	99.96
	Oven dry	Quinic acid	354.0942	C16 H18 O9	353.0869	2.723	-	95.95
		Nigerose	342.1156	C12 H22 O11	341.1083	1.499	-	97.26
		N,N-dimethyl-Safingol	329.3287	C20 H43 N O2	330.336	9.73	+	98
		9-Nonadecene	266.2969	C19 H38	284.3307	10.808	+	98.71
		Palmitic amide	255.2561	C16 H33 N O	256.2634	16.226	+	99.88
		Quinic acid	354.0942	C16 H18 O9	353.0869	2.723	-	95.95
	Shade dry	Nigerose	342.1156	C12 H22 O11	341.1083	1.499	-	97.26
		Adenine	135.0544	C5 H5 N5	134.0471	1.487	-	80.84
		9-Pentacosene	350.3907	C25 H50	368.4245	15.297	+	97.5
		Gravolenic acid	280.0941	C14 H16 O6	281.1014	8.281	+	98
		9-Tricosene	322.3592	C23 H46	340.393	13.644	+	95.87
Leaf	Freeze dry	6-Methoxyluteolin	316.0577	C16 H12 O7	315.0505	3.21	-	97.59
		Kaempferol 8-C-sulfate	366.0035	C15 H10 O9 S	364.9963	2.903	-	95.87
		Gossypetin 3-O-sulfate	397.9938	C15 H10 O11 S	442.992	4.732	-	91.55
		C17 Sphinganine	287.2822	C17 H37 N O2	288.2895	7.365	+	99.54

		C16 Sphinganine	273.2668	C16 H35 N O2	274.274	6.554	+	99.96
		Sphinganine	273.2668	C16 H35 N O2	274.274	6.554	+	99.96
	Oven dry	Kaempferol 8-C-sulfate	366.0035	C15 H10 O9 S	364.9963	2.903	-	95.87
		Apigenin 7-sulfate	350.0085	C15 H10 O8 S	349.0013	3.891	-	88.73
		7,8,3',4'-Tetrahydroxyflavone	286.0472	C15 H10 O6	285.0399	4.453	-	96.23
		Palmitic amide	255.2561	C16 H33 N O	256.2634	16.226	+	99.88
		1-Eicosene	280.3125	C20 H40	298.3463	11.144	+	98.08
		9-Pentacosene	350.3907	C25 H50	368.4245	15.297	+	97.5
	Shade dry	6-Methoxyluteolin	316.0577	C16 H12 O7	315.0505	3.21	-	97.59
		Kaempferol 8-C-sulfate	366.0035	C15 H10 O9 S	364.9963	2.903	-	95.87
		Gossypetin 3-O-sulfate	397.9938	C15 H10 O11 S	442.992	4.732	-	91.55
		Palmitic amide	255.2561	C16 H33 N O	256.2634	16.226	+	99.88
		1-Eicosene	280.3125	C20 H40	298.3463	11.144	+	98.08
		9-Pentacosene	350.3907	C25 H50	368.4245	15.297	+	97.5
Root	Freeze dry	Nigerose	342.1156	C12 H22 O11	341.1083	1.499	-	97.26
		Quercetin	302.042	C15 H10 O7	301.0347	2.201	-	94.92
		5Z-Caffeoylquinic acid	354.0942	C16 H18 O9	353.0869	2.723	-	95.95
		C17 Sphinganine	287.2822	C17 H37 N O2	288.2895	7.365	+	99.54
		C16 Sphinganine	273.2668	C16 H35 N O2	274.274	6.554	+	99.96
		Phytosphingosine	317.2928	C18 H39 N O3	318.3001	6.669	+	99.81
	Oven dry	Nigerose	342.1156	C12 H22 O11	341.1083	1.499	-	97.26

		D-Glucose	180.0633	C6 H12 O6	179.056	1.504	-	84.7
		DL-2-hydroxy stearic acid	300.2656	C18 H36 O3	299.2583	13.793	-	93.67
		9-Nonadecene	266.2969	C19 H38	284.3307	10.808	+	98.71
		Palmitic amide	255.2561	C16 H33 N O	256.2634	16.226	+	99.88
		1-Octadecanamine	269.3077	C18 H39 N	270.315	8.817	+	98.5
	Shade dry	Nigerose	342.1156	C12 H22 O11	341.1083	1.499	-	97.26
		Kaempferol 8-C-sulfate	366.0035	C15 H10 O9 S	364.9963	2.903	-	95.87
		9-Nonadecene	266.2969	C19 H38	284.3307	10.808	+	98.71
		Palmitic amide	255.2561	C16 H33 N O	256.2634	16.226	+	99.88
		Eudesmin	386.1728	C22 H26 O6	387.1802	8.277	+	99.84
